# Summer Clinical Oncology Research Experience (SCORE) Program: Engaging Undergraduates from Diverse Backgrounds in Cancer Research

**DOI:** 10.1007/s13187-022-02247-8

**Published:** 2023-01-13

**Authors:** Laura Liberman, Priya Singh, Kay See Tan, Ruth Gotian

**Affiliations:** 1 Office of Education and Faculty Affairs, Memorial Sloan Kettering Cancer Center (MSK), New York, NY USA; 2 Department of Epidemiology and Biostatistics, Memorial Sloan Kettering Cancer Center, New York, NY USA; 3 Department of Anesthesiology, Weill Cornell Medicine, New York, NY USA

**Keywords:** Cancer research, Student internship, Summer, Undergraduate, Training outcomes

## Abstract

Diversifying the future cancer research workforce requires that students engage in cancer research, persist in paths toward science, technology, engineering, mathematics, and medicine (STEMM) fields, and choose cancer research careers. The Summer Clinical Oncology Research Experience (SCORE) Program at Memorial Sloan Kettering, designed in 2010 to engage undergraduate (U) and post-baccalaureate (PB) students from diverse backgrounds in cancer research, is an 8-week summer program pairing an U or PB student with a faculty mentor to conduct cancer research. We report demographics and career paths for 2010–2019 SCORE students. Of 116 students, 112 (97%) attended public universities, and 75 (64%) were in their first 2 years of college. Race/ethnicity was Black/African American, 20 (17%); Hispanic/Latinx, 15 (13%); multiracial, five (4%); Asian, 40 (34%); White/Caucasian, 36 (31%). A total of 112 (97%) identified as female; 47 (41%) were first-generation college students, and 85 (73%) were from immigrant families. As of 2021, 114 (98%) persisted in paths toward STEMM careers: 44 (38%) medical school (MS) students, 14 (12%) residents, two (2%) practicing physicians, 12 (10%) pursuing non-MD STEMM advanced degrees, 21 (18%) working in non-MD STEMM fields, 17 (15%) applying to MS, and 4 (3%) U science majors. Cancer research participation significantly increased from 5% pre- to 84% post-SCORE. A total of 63/116 (54%) students subsequently co-authored 152 peer-reviewed publications, including 105 (69%) in oncology. SCORE engaged underrepresented U and PB students in cancer research, and 98% of these students persisted in paths toward STEMM careers. Long-term follow-up is needed to assess the enduring engagement of these underrepresented students in cancer research.

**Supplementary Information:**

The online version contains supplementary material available at 10.1007/s13187-022-02247-8.

## Introduction

Recent world events highlighting healthcare inequities demonstrate the need for an inclusive health workforce. In 2021, cancer was the second leading cause of US mortality, below heart disease and above COVID-19 [1]. In the 2022 US oncology workforce, < 36% identify as female, < 5% Hispanic/Latinx, 0.3% as Black/African American, and 0.1% American Indian/Alaska Native [2]. The cancer workforce must be diverse in gender identity, race/ethnicity, and other group identities, to reflect the population we serve [2, 3]. To join the cancer research workforce, students must engage in cancer research, persist in paths toward STEMM, and choose cancer research careers [4].

The Summer Clinical Oncology Research Experience (SCORE) Program was created in 2010 at Memorial Sloan Kettering Cancer Center (MSK) to engage U and PB students from diverse backgrounds in cancer research and to build a path for these students to persist in a direction toward STEMM careers. We reviewed the first decade of SCORE student demographics and career trajectories to assess SCORE’s progress in achieving program goals.

## Methods

### SCORE Description

SCORE is an 8-week summer cancer research program for U and PB students. The guiding principles of SCORE, inspired by a neighboring program designed to build a pathway for physician-scientists [5], include the creation of a nurturing community to minimize anxiety; verbal encouragement and role modeling by mentors and the Program Director (PD); research skill workshops providing a low-risk opportunity to try new things; transparent milestones with practice to foster performance accomplishments; and inclusion in team activities of the mentor/PI to enhance students’ sense of identity as a scientist.

We pair each SCORE student with a faculty physician or scientist at MSK to conduct mentored cancer research, either clinical or laboratory-based investigation. Students are trained in scientific presentation, abstract writing, and slide design; they participate in didactic lectures, career development workshops, and clinical observation. Students have weekly group lunches to share experiences, and they engage in group social activities. In weeks 6 and 7, respectively, students submit a 1-page abstract and PowerPoint slides for a 10-min presentation about their work. Week 8 includes 3 days of group rehearsals for oral presentations, an all-day SCORE Scientific Symposium, and a final debrief day to view the presentation recordings and provide peer feedback.

To be eligible, a student must be an U or PB enrolled in a degree- or certificate-granting program in the New York City area. We encourage applications from students who are early in their college careers or PBs, have limited research experience, or identify as being from groups underrepresented in medicine. SCORE was launched in 2010 by the MSK Program for Women Faculty Affairs; from 2010–2019, all student participants identified as female. As of 2019, SCORE was led by the MSK Office of Faculty Development (now the Office of Education and Faculty Affairs) and included students of varied gender identities. Due to our U54 NIH-funded partnership with the City University of New York (CUNY) and City College of New York (CCNY), SCORE primarily recruits from CUNY, a public university system with a racially, ethnically, and economically diverse student body.

Students complete an application with an essay, a transcript, and two recommendations. The PD solicits faculty interested in mentoring a summer student; prospective mentors must describe the project, defining the role of the student and expected outcomes. Based on student interests, experience, background, and other personal characteristics as evident in the essay and recommendations, the PD makes tentative mentor/mentee matches and invites the student to interview with the prospective mentor. The prospective mentor reviews student application materials, and the student reviews the project description and mentor biography. Matching is finalized after the interview if both mentor and mentee approve.

After SCORE, we encouraged students to submit their work to the Annual Biomedical Research Conference for Minority Students (ABRCMS), a National Institutes of Health-funded conference to promote students underrepresented in STEMM, held in various US cities. MSK supported ABRCMS participation; PD traveled with them to provide support, practice with students, and facilitate networking.

SCORE includes all current and prior SCORE students in an ongoing community of practice, which has been defined as “a persistent, sustaining social network of individuals who share and develop an overlapping knowledge base, set of beliefs, values, history, and experiences focused on a common practice” [6]. The PD maintains ongoing mentoring relationships with students, advising on extracurriculars, summer and gap year activities, and career planning; reviewing medical school or other application materials; writing recommendations; and engaging in mock interviews. Students maintain ongoing relationships with MSK faculty mentors and with SCORE alumni from all cohorts through social gatherings, educational events, emails, texts, and social media.

### Data Collection and Analysis

In this program evaluation, we retrospectively analyzed demographics and projects for SCORE students from 2010–2019. We determined career paths using a follow-up survey opened in 2020 and closed in 2021, to which 97/116 (84%) students responded (Online Resource 1). Median follow-up was 5 (range, 2–11) years. We also found follow-up data from Google, LinkedIn, and PubMed. We reviewed student applications and onboarding materials for age, school year, gender identity, race/ethnicity, country of origin, and other demographic factors. We reviewed the annual cost per student for 2018 and 2019. We summarized student demographic data and survey responses as frequencies (percentages) or medians (ranges). We compared proportions between groups using chi-square tests and summarized them as differences in proportions along with 95% Wald confidence intervals (CI). We conducted statistical analyses using R 3.5.1 (R Core Team, Vienna, Austria), and a two-sided *p* < 0.05 was considered significant.

## Results

### Student Demographics and Research Projects

**Fig. 1:**
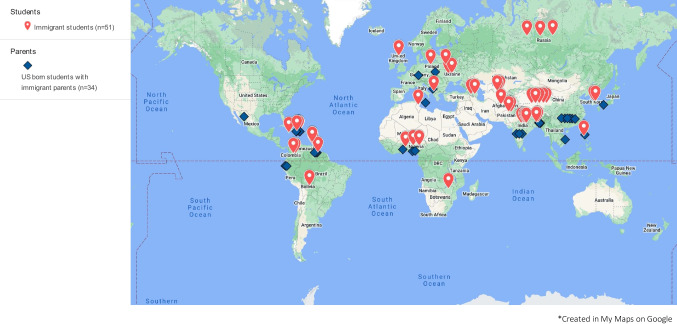
Country of origin of 85 MSK (New York, NY) SCORE 2010–2019 families who immigrated to the USA

In the first decade of SCORE, 116 students participated: 94 (81%), 21 (18%), and one (< 1%) participated for 1, 2, or 3 years, respectively. The median number of SCORE students annually was 16 (range, 4–22). Over this decade, these 116 students performed 130 mentored cancer research projects, 104 (80%) clinical and 26 (20%) laboratory, in 18 departments (Online Resource 2). At first participation, the median student age was 19 (range, 18–35) years. Demographic and other features are shown in Table 1. Most students were from public colleges and identified as female. In total, 64% were freshmen or sophomores; 14% were PBs. A total of 41% were the first generation (i.e., neither parent completed college), 34% were from racial/ethnic groups underrepresented in medicine, and 85 (73%) were from immigrant families from 37 countries in Asia, Europe, Latin America, the Caribbean, and Africa (Fig. 1, Online Resource 3).

**Table 1:** MSK (New York, NY) SCORE 2010–2019 Student Background Data (*n* = 116)

Feature	#	%
Public vs. Private College		
Public^a^	112	97%
Private	4	3%
Year in college		
Freshman	49	42%
Sophomore	26	22%
Junior	19	16%
Senior	6	5%
Post-baccalaureate (PB)	16	14%
Race/Ethnicity^b^		
Black/African American	20	17%
Hispanic/Latinx	15	13%
Multiracial	5	4%
Asian	40	34%
White/Caucasian	36	31%
Gender identity		
Female	112	97%
Male	4	3%
1st generation college student		
Yes	47	41%
No	69	59%
Origin		
Immigrant to the USA	51	44%
Child of immigrant parents	34	29%
Neither	31	27%

^a^These 112 students attended branches of City University of New York (CUNY), including Hunter College (84), City College of New York (27), and LaGuardia Community College (1)

^b^Underrepresented groups include Black/African American, Hispanic/Latinx, Multiracial

### Program Cost

The program cost was approximately $7000 per SCORE student for salary, program activities, and ABRCMS, covered through institutional funds, philanthropy, and the CCNY-MSK Partnership. The salary enabled students with limited economic resources to participate. We did not provide housing as all students were local.

### Follow-Up

Students submitted 95 projects to ABRCMS; all were accepted, for poster in 54 (57%) and oral in 41 (43%). Twenty (21%) ABRCMS presentations won awards, including 13/41 (32%) oral and 7/54 (13%) posters. The proportion of accepted presentations invited to be given orally was significantly higher among MSK submissions versus all ABRCMS submissions (41/95 = 43% vs. 1034/19,025 = 5%; difference = 38%, 95% CI 28–48%; *p* < 0.001). The award rate was also higher among MSK presentations versus all ABRCMS presentations (20/95 = 21% vs. 2617/19,025 = 14%; difference = 7%, 95% CI 0–16%; *p* < 0.001), as per personal communication in June 2021 with Leah Dixon, the Education Coordinator of ABRCMS.

As of 2021, 114 (98%) of 116 students persisted in paths toward STEMM careers. Of the 116 students, 93 (80%) were accepted/enrolled in medical school (MS), residents, practicing physicians, pursuing other advanced STEMM degree programs, or working in non-MD STEMM fields; 17 (15%) were applying to MS; and 4 (3%) advanced as U science majors (Table 2). A total of 16/16 (100%) PBs persisted in paths leading to STEMM careers, with 6 (38%) accepted/enrolled in MS, 5 (31%) in residency, 3 (19%) working healthcare providers, and one each pursuing a combined DVM/PhD degree or volunteering in STEMM while applying to MS.

**Table 2:** MSK (New York, NY) SCORE 2010–2019 student career trajectories (*n* = 116)

Current role	#	%
Current undergraduate	4	3%
Medical school		
Currently applying	17	15%
Accepted/enrolled^a^	44	38%
Graduated; in residency^b^	14	12%
Graduated; practicing physician^c^	2	2%
Other health/science advanced degree program (enrolled)^d^	12	10%
Working		
Non-MD healthcare provider^e^	5	4%
Other health/science field^f^	16	14%
Non-health/science	2	2%

^a^Includes 2 MD PhD, 2 MD MPH, 1 MD MBA

^b^Includes 5 medicine, 2 anesthesiology, 2 pediatrics, 2 surgery, 1 each in obstetrics/gynecology, psychiatry, and radiology

^c^Includes 1 attending radiologist and 1 attending radiation oncologist

^d^Includes 3 PhD, 2 Master’s (1 MPH, 1 Data Science), 2 physician assistant (PA), 2 podiatry, 1 doctor of veterinary medicine (DVM)/PhD, 1 occupational therapy (OT), 1 nursing

^e^Includes 3 dentists, 1 nurse, 1 nurse practitioner

^f^Includes 8 biomedical research, 5 healthcare administration, 2 public health, 1 software engineer

SCORE also served as an effective recruitment strategy for MSK. A total of 47 (41%) of 116 students were later employed by MSK in a different program either for summer(s) (11) or longer (36). Of the latter 36 students, 35 worked at MSK as lab technicians (14), research assistants/coordinators (12), or other roles (9); one returned for fellowship at MSK and is now a faculty physician at MSK.

### Subsequent Participation in Cancer Research

Before SCORE, 6/116 (5%) students had experience in cancer research; after SCORE, 97/116 (84%) continued cancer research participation (difference = 78%, 95% CI 71–86%; *p* < 0.001). None of the students had scientific publications in PubMed before SCORE. After SCORE, 63 (54%) students authored 152 unique peer-reviewed publications, of which 105 (69%) related to oncology. These 63 students published a median of 1 paper each (average, 2.5; range, 1–20). Of 152 papers, 45 (30%) reported work the student did with their SCORE mentor. The student was the first author on 24/152 (16%) papers and on 13/105 (12%) papers in oncology (Online Resource 4).

## Discussion

Student engagement in cancer research and persistence in paths toward STEMM careers are necessary steps to diversify the future cancer research workforce. Persistence in STEMM is impacted by a complex interplay of influences, including (1) self-efficacy—confidence in one’s ability to do scientific research—which is informed by the emotional state while performing the task (a relaxed/low anxiety state is most conducive), verbal support, role models, and performance accomplishments; and (2) science identity—a sense of belonging as a scientist [4, 7–10]. SCORE’s guiding principles align with these influences that enhance persistence in STEMM.

In the first decade of SCORE, most students attended public universities, identified as female, and were from immigrant families; over 40% were first-generation college students; one-third were from racial/ethnic groups underrepresented in oncology. Our students’ demographic features align with the call to reduce bias in American medicine based on features including race, ethnicity, gender, and immigration status [11]. The robust participation of women in SCORE is encouraging, since women are underrepresented in cancer research [12, 13]. After SCORE, most students did further cancer research and published scientific papers, two-thirds of which were in oncology. These findings show that at a median of a 5-year follow-up, SCORE engaged diverse students in cancer research.

Publication after summer research programs has been used as an outcome measure for program value [14]. The 54% publication rate after SCORE for our students, primarily undergraduates, is within the 23–68% range reported after summer programs for more advanced medical or graduate students [15, 16]. 2/3 of students’ publications after SCORE were authored with a new mentor, similar to prior results from a summer medical student program [14]. We believe that this finding suggests that SCORE may increase our students’ desirability as mentees as well as enhance their self-efficacy and science identity, building their confidence to seek these new opportunities.

Early engagement of undergraduates in SCORE increases their chances of gaining mentors and more research opportunities and aligns with suggested interventions to increase persistence in STEMM [10]. Early college students may have difficulty finding a research position without research experience, and they cannot get research experience without a research position. To break this cycle, we seek projects accessible to motivated students who lack research experience, considering prior coursework when matching students to projects. We also reach out to PBs who often have limited research experience; all our PBs persisted in paths toward STEMM careers.

ABRCMS participation allowed students to present at a national meeting in a nurturing environment, get positive feedback, interact with peers and mentors from similar backgrounds, network with program representatives, and expand their community of practice. In prior work, ABRCMS participation increases research self-efficacy, sense of belonging, and the likelihood of pursuing research careers [17]. The SCORE community of practice provides a supportive network with ongoing mentoring and connection with PD, peers from all SCORE cohorts, and MSK faculty, as per the previously described “affirming kindness and community” and “learning community” [8, 9].

A total of 98% of SCORE students persisted in paths toward STEMM careers. We hypothesize that SCORE fosters this outcome via a nurturing environment, early engagement, individual and peer mentoring, verbal encouragement, skill-building, transparent milestones, team activities, success opportunities, and an ongoing community of practice—aligning with factors enhancing student persistence in STEMM, such as self-efficacy and science identity [4, 7–10].

Our data must be interpreted in the context of the study design. Our NY cancer center may not be a widely representative setting. Some alumni career paths remain undefined. We did not ask about students’ identity as cancer researchers or their intention to pursue careers in cancer research; this issue could be addressed in future surveys. We cannot prove that SCORE caused our outcomes; the selection process may have identified individuals destined to succeed.

## Conclusion

The SCORE program engaged diverse U and PB students in cancer research, and 98% persisted in paths toward STEMM careers. These steps are necessary but not sufficient to diversify the cancer research workforce. Follow-up is necessary to assess the long-term participation of these diverse students in cancer research. Further attention must be paid to specific groups underrepresented in cancer research, such as Black men; to the interplay of factors such as gender, race, and ethnicity in mentoring relationships; and to enhance cultural diversity awareness for mentors in STEMM [18–20]. Additional work is needed to enhance cancer research workforce diversity in all aspects of identity, including gender identity, race/ethnicity, sexual orientation, and ability/disability status.

## Supplementary Information

Below is the link to the electronic supplementary material.

Supplementary file1 (PDF 1545 KB)
