# Supplementary material for: Summer Clinical Oncology Research Experience (SCORE) Program: Engaging Undergraduates from Diverse Backgrounds in Cancer Research
Source: J Cancer Educ. 2023 Jan 13;38(4):1187–92. doi: 10.1007/s13187-022-02247-8 (PMC9836916; doi:10.1007/s13187-022-02247-8)
Supplement: Supplementary file 1 — Supplementary file1 (PDF 1545 KB) [file 13187_2022_2247_MOESM1_ESM.pdf]

Online Resource 1

MSK Summer Clinical Oncology Research Experience (SCORE) 2010-2019 Students Follow-up Survey

1. Last Name

2. First Name

3. Permanent Email

4. Current Phone Number

5. Please indicate your current position.

- Undergraduate student
- Post-baccalaureate student
- Medical student (MD or DO Program)
- Intern or Resident (after Medical School)
- Fellow (after Residency)
- Graduate student (Master's or PhD program)
- Nursing student
- PA Student
- Working as a physician after medical school
- Working as a physician-scientist
- Working as a scientist
- Working in health or science field (non-physician, non-scientist)
- Working in a field other than health or science
- Taking a gap year
- Other (describe below)

Please give details about what you are doing now and where.

6. Have you done any research outside of class since SCORE?

- Cancer research
- Non-cancer research
- Both cancer and non-cancer research
- Have not done research

Please briefly explain your research. If ?1 experience, please provide a numbered list.

.

7. Have you done oncology non-research activities since SCORE? (eg clinical care, shadowing...)

- Yes
- No

If so, what/when/where?

8. Would you recommend SCORE to other students?

- Yes
- No

How has SCORE impacted your career path?

9. What is the highest degree you have achieved?

- High School Diploma
- BA
- BS
- BSN
- MA
- MS
- MPH
- MSN
- PhD
- DO
- MD

Other (what)? If Bachelor's, Master's or PhD, in what subject? If dual-degree, then which ones?

10. Undergraduate School Information

School name and location (city, state)

If graduated, give graduation year, degree, major

If undergraduate, provide current grade, expected graduation year, expected degree, and major

11. Which of the following describes you, if any? (check all that apply)

- Medical school (MD or DO); applying or plan to apply
- Medical school (MD or DO); accepted/enrolled
- Medical school (MD or DO); graduated
- Other health or science degree program; applying or plan to apply
- Other health or science degree program; accepted/enrolled
- Other health or science degree program; graduated
- Non-science/non-health graduate program; applying or plan to apply
- Non-science/non-health graduate program; accepted/enrolled
- Non-science/non-health graduate program; graduated
- Taking a gap year
- Took one or more gap years after college, now in school

Other (please specify). If not MD or DO, what field of study or work?

12. Advanced Degree Program Information - Medical, Nursing, MPH, PA... (if accepted/enrolled or graduated)

School name and location (city, state)

If graduated, provide graduation year and degree received (eg MD, DO, PhD, etc.)

If now advanced degree student, give expected graduation year and degree (eg MD, DO, PhD)

What subject are you studying/did you study?

Start date (year)

13. How many gap years, if any, have you taken?

- 0
- 1
- 2
- 3+

What did you do and where?

14. In how many published papers, if any, have you been a co-author?

- 0
- 1
- 2
- 3
- 4+

Please give numbered list of publications (authors, title, journal, year, volume, pages start-end)

15. Have you worked at MSK since SCORE?

- Worked at MSK in the past
- Currently working at MSK
- Never worked at MSK

If yes, please indicate your position, when you worked (Was it during a gap year? What are the start-finish dates, in mo/yr format, you worked?) and give a brief description of what you did/do.

16. Please attach updated CV Word Document, format: First Name Last Name CV Date

17. Other comments?

THANK YOU! WE LOVE TO HEAR FROM OUR SCORE FAMILY!

Online Resource 2. Research Projects Conducted by 116 MSK SCORE Students, 2010-2019

School Year[a]	Department(s)	Project Title
1	Anesthesiology and Critical Care Medic	A double-masked placebo-controlled clinical trial of postoperative opioid-sparing effects of intraoperative dexmedetomidine infusion for video-assisted thoracic surgery
1	Anesthesiology and Critical Care Medic	How changes in mean arterial pressure during non-cardiac thoracic surgery affect intraoperative urinary output and postoperative renal function: A retrospective data review
1	Cancer Biology and Genetics	The role of fatty acids in crosstalk between melanoma cells and the microenvironment
1	Cancer Biology and Genetics	Understanding the crosstalk between melanoma cells and the microenvironment using a chemical screening approach
2	Cancer Biology and Genetics	Characterizing chemicals that modulate metastasis in the zebrafish
2	Cancer Biology and Genetics	Investigating the role of Y-family polymerases in stress-induced mutagenesis and drug resistance
2	Cancer Biology and Genetics	Multicolor imaging of metastasis using zebrafish models of melanoma
2	Chemical Biology	Impacts of methylglyoxal (MGO) on epigenetic regulation
2	Developmental Biology	Applying mosaic mutant analysis with spatial and temporal control of recombination (MASTR) to study sonic hedgehog signaling in adult neural stem cells
1	Epidemiology and Biostatistics	Digital data extraction and statistical methodology research to evaluate predictive cancer biomarkers
1	Epidemiology and Biostatistics	Fundamental contributions of statistics to cancer screening & intervention
1	Epidemiology and Biostatistics	Diabetes and endometrial cancer risk: a literature review
2	Epidemiology and Biostatistics	Validity of self-reported data in women with breast cancer
3	Epidemiology and Biostatistics	The landscape of cancer in India
1	HOPP[b], Neurology	Reactive A1 astrocytes promote cancer growth within the leptomeninges
2	HOPP, Medicine	Influence of Stag2 mutations on activating histones: A mechanism for myeloid malignancies?
PB	HOPP, Medicine	Do Stag2 cohesin mutations cause myeloid leukemia?
PB	Laboratory Medicine	Clinical and laboratory characterization of coronaviruses infection in cancer patients
1	Medical Physics	Monitoring of patient movement during external beam radiotherapy for prostate cancer
1	Medical Physics, Pathology	Diffusion-weighted (DW) Magnetic Resonance Imaging (MRI) based apparent diffusion coefficient (ADC) value assessment in mapping the microscopic extent of in situ or invasive breast cancer
3	Medical Physics	4DMRI and respiratory motion assessment for radiotherapy planning
3	Medical Physics	Reducing error and increasing consistency in the segmentation of anatomical structures for radiotherapy planning
1	Medicine	Neem tree: A review of the literature on a popular dietary supplement
1	Medicine	Sutherlandia (Lessertia Frutescens): Evidence for medicinal use of a traditional herbal remedy
1	Medicine	Post-operative delirium and other surgical outcomes in the oldest old cancer patients
1	Medicine	Impact of trastuzumab-induced cardiotoxicity and subsequent trastuzumab interruption on breast cancer outcomes
1	Medicine	PTEN status and response to androgen receptor targeted therapy in prostate cancer
1	Medicine	The role of massage therapy in symptom reduction and quality of life
1	Medicine	Ultrasound: A modern tool in assessing muscle quality
1	Medicine	Surveillance of clostridium difficile infection in elderly patients with cancer
1	Medicine	Where are they now? Tri-institutional MD PhD follow-up at 40 Years
1	Medicine	Extreme jobs: Implications for women doctors and scientists in oncology
1	Medicine	Colonoscopy and polypectomy in patients younger than 50 years old: Indications and findings
1	Medicine	Screening of upper gastrointestinal cancers in patients with Lynch Syndrome: Guidelines and pilot survey of current practice
1	Medicine	National guidelines for surveillance testing in patients with solid tumors: Variation and specificity
1	Medicine	Correlating patient outcomes and characteristics with occurrence of genetic mutations in peripheral T-cell lymphomas treated with histone deacetylase inhibitors
1	Medicine	Application of the Nielson criteria to predict response to bicalutamide in women with androgen receptor positive, triple negative metastatic breast cancer
2	Medicine	A review of the utility of cardiac stress testing among patients undergoing pre-hematopoietic stem cell transplantation screening
2	Medicine	Integrative medicine outpatient therapies: Survey of 2013 Utilization
2	Medicine	Genomic alterations in prostate cancer patients treated with hormones and radiation predict response and resistance
2	Medicine	Agreement is high across duplicate patient reported outcome (PRO) pesponses
2	Medicine	Use of mitotane in adrenocortical carcinoma: effectiveness and risk
2	Medicine	Enhancing diversity among physician-scientists: Gateways to the Laboratory
2	Medicine	Late effects of autologous stem cell transpantation (ASCT) in elderly patients with Lymphoma
2	Medicine	Development of chimeric antigen receptor (CAR) T cell therpy for multiple myeloma
2	Medicine	Patient financial responsibility for oral anti-neoplastic drugs
2	Medicine	A review of serum magnesium levels in patients with metastatic breast cancer receiving pertuzumab
3	Medicine	Dual mTOR inhibitor MLN0128 in patients with metastatic castration-resistant prostate cancer (mCRPC)
3	Medicine	The effect of hormonal therapy prior to prostatectomy in patients with high risk prostate cancer
3	Medicine	Develop modified vaccinia virus Ankara (MVA)-based immunotherapy for cancers
3	Medicine	Are there better ways to give combination immunotherapy for melanoma that have less side effects?
3	Medicine	Clinical and immunological variables associated with benefit from combination immune checkpoint blockade in melanoma
3	Medicine	Risk factors for the development of young adult colorectal cancer
PB	Medicine	A popular botanical called "field bindweed"-- Any clinical value?
PB	Medicine	Evaluation of wellness workshop tailored to MSK Environmental Service employees
PB	Medicine	Development of dual target chimeric antigen receptor (CAR) T cell therapy for multiple myeloma
PB	Medicine	Integrative medicine approaches to reduce chemotherapy-induced peripheral neuropathy severity
1	Medicine	Integrative oncology
2	Medicine, Radiology	Integrative medicine therapies reduce breast biopsy-associated anxiety
2	Medicine, Surgery, Nursing	Fertility preservation after cancer treatment: Educating teens about options
3	Molecular Biology	Regulatory mechanisms of a primary genome protecting complex
1	Neurology	The cerebrospinal fluid circulating tumor cells (CSF CTC) test is sensitive and specific in diagnosing leptomeningeal metastases and can predict survival
1	Neurology	Natural history of spinal metastases in breast cancer
1	Neurology	Safety of physical & occupational therapy interventions for patients with hematological cancers and severe thrombocytopenia
1	Neurology	Spine bracing in the oncologic population
2	Neurology	Educating patients about services available at MSK: Multidisciplinary (MDC) Clinic Approach to Optimize Care for Frail Patients with Central Nervous System Cancers

3	Neurology	Evaluation of circulating tumor cells for diagnosis of leptomeningeal metastasis from solid tumors
3	Neurology	The likelihood of developing leptomeningeal metastasis in cancer patients with neurological symptoms but negative neuroimaging and cerebrospinal fluid cytology
3	Neurology	Compliance with physical therapy and exercise in men with prostate cancer and spinal metastases is associated with relief of back pain
3	Neurology	Safety of physical therapy in lymphoma patients with thrombocytopenia
1	Pathology	Determinants of chemoresistance in acute myelogeous leukemia (AML)
1	Pathology	Detection of novel translocations by next generation RNA sequencing of pediatric undifferentiated/primitive sarcomas
2	Pathology	Investigating the role of translation in acute myeloid leukemia
2	Pathology	The 21-gene recurrence score in ER(+), HER2(-) breast cancer patients age 70 and older
3	Pathology	Role of MYC in squamous differentiation of pancreatic ductal adenocarcinoma
3	Pathology	Size information in cell-free DNA (cfDNA) can predict tumor fraction in multiple solid tumors and blood cancers
4	Pathology	Clonal evolution in cancer
PB	Pathology	ClonTracer enables monitoring of dynamic clonal changes in pancreatic ductal adenocarcinoma
PB	Pathology	Immunohistochemical analysis of biomarkers identified by genomic analysis in renal cell carcinomas
1	Pathology	Surgical pathology of microinvasive breast cancer
1	Pathology, Medical Physics	Breast cancer variability: Assessment with histogram analysis using diffusion weighted Imaging
2	Pathology, Radiology	Using diffusion-weighted imaging (DWI) magnetic resonance (MR) to predict the presence of breast cancer at margins
1	Pediatrics	Correlating dosimetry with survival in pediatric patients treated with intrathecal radioimmunotherapy for relapsed CNS malignancies
2	Pediatrics	Is it really RB? Late metastases in patients with a history of retinoblastoma
4	Pediatrics	Intrathecal radioimmunotherapy for pediatric neuroblastoma
2	Pediatrics, Nursing	MYCN as an indicator of survival and relapse in central nervous system (CNS) neuroblastoma patients
PB	Pediatrics, Nursing	Assessing CSF flow dynamics in pediatric patients with CNS tumors treated with intraventricular radioimmunotherapy
1	Psychiatry and Behavioral Sciences	Tobacco use and lung cancer among Chinese livery drivers
1	Psychiatry and Behavioral Sciences	The impact of patient-physician language concordance on quality of care and outcomes
1	Psychiatry and Behavioral Sciences	Framing smokeless tobacco control messages for South Asians
1	Psychiatry and Behavioral Sciences	Stories from head & neck cancer survivors: A content analysis
1	Psychiatry and Behavioral Sciences	Depression in older cancer survivors
2	Psychiatry and Behavioral Sciences	Edtech-HPV: A community approach using education and technology to increase HPV vaccination
2	Psychiatry and Behavioral Sciences	The Taxi Network: Decreasing cancer risk through community-based participatory research
2	Psychiatry and Behavioral Sciences	Impact of language barriers on access to and receipt of cancer genetic counseling in Limited English Proficiency (LEP) patients
2	Psychiatry and Behavioral Sciences	Health and cancer disparities experienced by sexual and gender minority (SGM) People: A scoping review
3	Psychiatry and Behavioral Sciences	HPV vaccination rates in Mexican/Mexican American children are lower in boys than girls
3	Psychiatry and Behavioral Sciences	Assessing the Impact of the Integrated Cancer Care Access Network (ICCAN) on adherence to radiotherapy appointments: A pilot study
3	Psychiatry and Behavioral Sciences	Factors contributing to end of life and bereavement outcomes among caregiving families of advanced cancer patients
4	Psychiatry and Behavioral Sciences	Assessing the spiritual needs and well-being of Latino cancer patients
PB	Psychiatry and Behavioral Sciences	Putting the test to the test: A new way to detect false fluency among physicians
PB	Psychiatry and Behavioral Sciences	Colorectal cancer screening in the Mexican community
PB	Psychiatry and Behavioral Sciences	Tanning selfies posted on Instagram: A critical analysis using appearance management and perception theories
2	Radiation Oncology	Is radiation therapy useful to treat osseous metastases from rhabdomyosarcoma?
3	Radiation Oncology	The effect of young age and biologic subtype in breast cancer
PB	Radiation Oncology	Radiation therapy for early glottic carcinoma: The MSK experience
1	Radiology	Survival following minimally invasive treatment of hepatocellular carcinoma
1	Radiology	Is the incidence of cholangitis different when biliary stents are placed above vs. across the papilla?
1	Radiology	Preparation of ^{89}Zr-labeled adenovirus for cancer imaging
1	Radiology	Imaging of neuroblastoma with ^{123}I- MIBG scintigraphy and ^{18}F-FDG PET
1	Radiology	Texture analysis to predict survival in patients with glioblastomas receiving Immunotherapy
1	Radiology	Histological confirmation of B-cell lymphoma Infiltration of multiple tissues in a mouse model
1	Radiology	AZD2281-FL: A novel imaging agent for intraoperative imaging and surgical resection of brain tumors
2	Radiology	Radiogenomics in ovarian cancer: linking phenotype with genotype
3	Radiology	Does age make a difference? Mammogram findings in women 80 years of age and older
3	Radiology	Studying cancer metabolism using hyperpolarized MRI
3	Radiology	Design of ^{18}F-receptor tyrosine kinase inhibitors for glioblastoma diagnosis
4	Radiology	^{18}F-FDG PET/CT for systemic staging of newly diagnosed breast cancer in men
4	Radiology	^{18}F-FDG PET/CT staging for breast cancer in women younger than 40 years old
4	Radiology	Clinical outcomes following mixed treatment response as measured on FDG PET/CT
PB	Radiology	Breast Imaging Reporting and Data System (BI-RADS) subcategories 4A/4B/4C: Frequency and cancer rate
PB	Radiology	Communication skills for breast imaging
2	Radiology, Surgery	Applying texture analysis for predicting malignancy in pancreatic cysts
1	Surgery	Trismus after radiation therapy for head and neck cancer
1	Surgery	The role of adjuvant chemotherapy in patients with residual nodal disease after neoadjuvant chemoradiation and ssophagectomy
2	Surgery	Factors associated with prolonged survival in patients with esophageal cancer
2	Surgery	Outcomes of minimally invasive esophagectomy for esophageal cancer
2	Surgery	Efficacy and toxicity of intravitreal melphalan with concomitant ophthalmic artery chemosurgery for retinoblastoma
PB	Surgery	A second chance: Measuring women's quality of life after burn surgery in developing countries
PB	Surgery	Breast questionnaire for women who have suffered burns

[a]1=freshman, 2=sophomore, 3=junior, 4=senior, PB=post-baccalaureate

[b]HOPP = Human Oncology and Pathogenesis Program

Online Resource 3. Raw Geographic Data for Country-of-Origin Map (Fig. 1) of 85 MSK SCORE 2010-2019 Families Who Immigrated to the U.S.

Country	Immigrant Students (n=51)	US born students (n=34), Immigrant parents (n=35)[a]
**ASIA**	**22**	**17**
Afghanistan	1	
Bangladesh	3	3
China	5	6
India	5	3
Japan		1
Pakistan	2	
Philippines	1	1
South Korea	2	
Taiwan		1
Uzbekistan	3	1
Vietnam		1
**EUROPE**	**12**	**4**
Albania	1	
Azerbijan	1	
Armenia	1	
Belarus	1	
Germany		1
Malta		1
Montenegro		1
Poland	1	1
Russia[b]	3	
UK	1	
Ukraine	3	
**LATIN AMERICA**	**6**	**6**
Bolivia	1	
Columbia	2	
DR	3	3
Ecuador		2
Mexico		1
**CARIBBEAN**	**6**	**5**
Grenada	2	
Guyana	1	3
Haiti	1	2
Jamaica	1	
Trinidad	1	
**AFRICA**	**5**	**3**
Ghana		1
Nigeria	2	2
Togo	1	
Tunisia	1	
Zimbabwe	1	

[a]1 student had mother from Germany and father from Ghana

[b]Russia is transcontinental (Europe and Asia); included with Europe

Online Resource 4. 152 Peer-Reviewed Papers by MSK SCORE (2010-2019) Students After Program (PubMed or accepted as of 09/28/2021)

CLICK ON TITLES FOR HYPERLINK TO PAPERS

Title	Reference
**AGARONNIK, NICOLE**	
*Perspectives of Patients with Pre-existing Mobility Disability on the Process of Diagnosing Their Cancer.	**Agaronnik ND**, El-Jawahri A, Iezzoni LI. J Gen Intern Med. 2021 May;36(5):1250-1257. doi: 10.1007/s11606-020-06327-7. Epub 2020 Nov 17. PMID: 33205226; PMCID: PMC8131437.
*Exploring attitudes about developing cancer among patients with pre-existing mobility disability.	**Agaronnik ND**, El-Jawahri A, Iezzoni LI. Psychooncology. 2021 Apr;30(4):478-484. doi: 10.1002/pon.5574. Epub 2020 Oct 25. PMID: 33064885; PMCID: PMC8026657.
*Exploring Cancer Treatment Experiences for Patients With Preexisting Mobility Disability.	**Agaronnik ND**, El-Jawahri A, Kirschner K, Iezzoni LI. Am J Phys Med Rehabil. 2021 Feb 1;100(2):113-119. doi: 10.1097/PHM.0000000000001622. PMID: 33065580; PMCID: PMC7855292.
Accommodating patients with obesity and mobility difficulties: Observations from physicians.	**Agaronnik ND**, Lagu T, DeJong C, Perez-Caraballo A, Reimold K, Ressalam J, Iezzoni LI. Disabil Health J. 2021 Jan;14(1):100951. doi: 10.1016/j.dhjo.2020.100951. Epub 2020 Jun 26. PMID: 32723692; PMCID: PMC7762732.
*Exploring the Process of Cancer Care for Patients With Pre-Existing Mobility Disability.	**Agaronnik ND**, El-Jawahri A, Lindvall C, Iezzoni LI. JCO Oncol Pract. 2021 Jan;17(1):e53-e61. doi: 10.1200/OP.20.00378. Epub 2020 Dec 22. PMID: 33351675; PMCID: PMC8257981.
*Use of Natural Language Processing to Assess Frequency of Functional Status Documentation for Patients Newly Diagnosed With Colorectal Cancer.	**Agaronnik N**, Lindvall C, El-Jawahri A, He W, Iezzoni L. JAMA Oncol. 2020 Oct 1;6(10):1628-1630. doi: 10.1001/jamaoncol.2020.2708. PMID: 32880603; PMCID: PMC7489406.
*Challenges of Developing a Natural Language Processing Method With Electronic Health Records to Identify Persons With Chronic Mobility Disability.	**Agaronnik ND**, Lindvall C, El-Jawahri A, He W, Iezzoni LI. Arch Phys Med Rehabil. 2020 Oct;101(10):1739-1746. doi: 10.1016/j.apmr.2020.04.024. Epub 2020 May 21. PMID: 32446905; PMCID: PMC7529728.
Accessibility of Medical Diagnostic Equipment for Patients With Disability: Observations From Physicians.	**Agaronnik N**, Campbell EG, Ressalam J, Iezzoni LI. Arch Phys Med Rehabil. 2019 Nov;100(11):2032-2038. doi: 10.1016/j.apmr.2019.02.007. Epub 2019 Mar 25. PMID: 30922882; PMCID: PMC6761045.
Exploring issues relating to disability cultural competence among practicing physicians.	**Agaronnik N**, Campbell EG, Ressalam J, Iezzoni LI. Disabil Health J. 2019 Jul;12(3):403-410. doi: 10.1016/j.dhjo.2019.01.010. Epub 2019 Feb 1. PMID: 30765256; PMCID: PMC6581576.
Communicating with Patients with Disability: Perspectives of Practicing Physicians.	**Agaronnik N**, Campbell EG, Ressalam J, Iezzoni LI. J Gen Intern Med. 2019 Jul;34(7):1139-1145. doi: 10.1007/s11606-019-04911-0. Epub 2019 Mar 18. PMID: 30887435; PMCID: PMC6614249.
Musical Chairs: Using Wheelchair Ballroom Dance in Disability Education.	**Agaronnik N**. JAMA. 2018 Jul 3;320(1):14-15. doi: 10.1001/jama.2018.8081. PMID: 29971376.
*Prospective comparison of the accuracy of the New England Spinal Metastasis Score (NESMS) to legacy scoring systems in prognosticating outcomes following treatment of spinal metastases.	Schoenfeld AJ, Ferrone ML, Blucher JA, **Agaronnik N**, Nguyen L, Tobert DG, Balboni TA, Schwab JH, Shin JH, Sciubba DM, Harris MB. Spine J. 2022 Jan;22(1):39-48. doi: 10.1016/j.spinee.2021.03.007. Epub 2021 Mar 16. PMID: 33741509; PMCID: PMC8443703.
Use of Accessible Weight Scales and Examination Tables/Chairs for Patients with Significant Mobility Limitations by Physicians Nationwide.	Iezzoni LI, Rao SR, Ressalam J, Bolcic-Jankovic D, Donelan K, **Agaronnik N**, Lagu T, Campbell EG. Jt Comm J Qual Patient Saf. 2021 Oct;47(10):615-626. doi: 10.1016/j.jcjq.2021.06.005. Epub 2021 Jun 23. PMID: 34364797; PMCID: PMC8464497.
Natural language processing for prediction of readmission in posterior lumbar fusion patients: which free-text notes have the most utility?	Karhade AV, Lavoie-Gagne O, **Agaronnik N**, Ghaednia H, Collins AK, Shin D, Schwab JH. Spine J. 2021 Aug 15:S1529-9430(21)00852-4. doi: 10.1016/j.spinee.2021.08.002. Epub ahead of print. PMID: 34407468.
*Natural Language Processing to Identify Advance Care Planning Documentation in a Multisite Pragmatic Clinical Trial.	Lindvall C, Deng CY, Moseley E, **Agaronnik N**, El-Jawahri A, Paasche-Orlow MK, Lakin JR, Volandes A, Tulsky TAIJA. J Pain Symptom Manage. 2021 Jul 14:S0885-3924(21)00428-0. doi: 10.1016/j.jpainsymman.2021.06.025. Epub ahead of print. PMID: 34271146.
*Associations Between Disability and Breast or Cervical Cancers, Accounting for Screening Disparities.	Iezzoni LI, Rao SR, **Agaronnik ND**, El-Jawahri A. Med Care. 2021 Feb 1;59(2):139-147. doi: 10.1097/MLR.0000000000001449. PMID: 33201087; PMCID: PMC7855335.
Physicians' Perceptions Of People With Disability And Their Health Care.	Iezzoni LI, Rao SR, Ressalam J, Bolcic-Jankovic D, **Agaronnik ND**, Donelan K, Lagu T, Campbell EG. Health Aff (Millwood). 2021 Feb;40(2):297-306. doi: 10.1377/hlthaff.2020.01452. PMID: 33523739; PMCID: PMC8722582.
Reducing an entrée portion size does not affect the amount of dessert consumed.	Levitsky D, **Agaronnik N**, Zhong W, Morace C, Barre L, Michael JJ. Appetite. 2020 Aug 1;151:104684. doi: 10.1016/j.appet.2020.104684. Epub 2020 Mar 29. PMID: 32234533.
*Cross-Sectional Analysis of the Associations Between Four Common Cancers and Disability.	Iezzoni LI, Rao SR, **Agaronnik ND**, El-Jawahri A. J Natl Compr Canc Netw. 2020 Aug;18(8):1031-1044. doi: 10.6004/jnccn.2020.7551. PMID: 32755976; PMCID: PMC8715389.
Quantifying the imprecision of energy intake of humans to compensate for imposed energetic errors: A challenge to the physiological control of human food intake.	Levitsky DA, Sewall A, Zhong Y, Barre L, Shoen S, **Agaronnik N**, LeClair JL, Zhuo W, Pacanowski C. Appetite. 2019 Feb 1;133:337-343. doi: 10.1016/j.appet.2018.11.017. Epub 2018 Nov 23. PMID: 30476522.
**ALIJA, BESMIRA**	
*Cohesin Members Stag1 and Stag2 Display Distinct Roles in Chromatin Accessibility and Topological Control of HSC Self-Renewal and Differentiation.	Viny AD, Bowman RL, Liu Y, Lavallée VP, Eisman SE, Xiao W, Durham BH, Navitski A, Park J, Braunstein S, **Alija B**, Karzai A, Csete IS, Witkin M, Azizi E, Baslan T, Ott CJ, Pe'er D, Dekker J, Koche R, Levine RL. Cell Stem Cell. 2019 Nov 7;25(5):682-696.e8. doi: 10.1016/j.stem.2019.08.003. Epub 2019 Sep 5. PMID: 31495782; PMCID: PMC6842438.
**AZZOPARDI, STEPHANIE**	
*p53 and p16Ink4a/p19Arf Loss Promotes Different Pancreatic Tumor Types from PyMT-Expressing Progenitor Cells.	**Azzopardi S**, Pang S, Klimstra DS, Du YN. Neoplasia. 2016 Oct;18(10):610-617. doi: 10.1016/j.neo.2016.08.003. Epub 2016 Sep 21. PMID: 27664376; PMCID: PMC5035259.

Risk of Zika microcephaly correlates with features of maternal antibodies.	Robbiani DF, Olsen PC, Costa F, Wang Q, Oliveira TY, Nery N Jr, Aromolaran A, do Rosário MS, Sacramento GA, Cruz JS, Khouri R, Wunder EA Jr, Mattos A, de Paula Freitas B, Sarno M, Archanjo G, Daltro D, Carvalho GBS, Pimentel K, de Siqueira IC, de Almeida JRM, Henriques DF, Lima JA, Vasconcelos PFC, Schaefer-Babajew D, **Azzopardi SA**, Bozzacco L, Gazumyan A, Belfort R Jr, Alcântara AP, Carvalho G, Moreira L, Araujo K, Reis MG, Keesler RI, Coffey LL, Tisoncik-Go J, Gale M Jr, Rajagopal L, Adams Waldorf KM, Dudley DM, Simmons HA, Mejia A, O'Connor DH, Steinbach RJ, Haese N, Smith J, Lewis A, Colgin L, Roberts V, Frias A, Kelleher M, Hirsch A, Streblow DN, Rice CM, MacDonald MR, de Almeida ARP, Van Rompay KKA, Ko AI, Nussenzweig MC. J Exp Med. 2019 Oct 7;216(10):2302-2315. doi: 10.1084/jem.20191061. Epub 2019 Aug 14. PMID: 31413072; PMCID: PMC6781003.
A Combination of Two Human Monoclonal Antibodies Prevents Zika Virus Escape Mutations in Non-human Primates.	Keeffe JR, Van Rompay KKA, Olsen PC, Wang Q, Gazumyan A, **Azzopardi SA**, Schaefer-Babajew D, Lee YE, Stuart JB, Singapuri A, Watanabe J, Usachenko J, Ardeshir A, Saeed M, Agudelo M, Eisenreich T, Bournazos S, Oliveira TY, Rice CM, Coffey LL, MacDonald MR, Bjorkman PJ, Nussenzweig MC, Robbiani DF. Cell Rep. 2018 Nov 6;25(6):1385-1394.e7. doi: 10.1016/j.celrep.2018.10.031. PMID: 30403995; PMCID: PMC6268006.
Recurrent Potent Human Neutralizing Antibodies to Zika Virus in Brazil and Mexico.	Robbiani DF, Bozzacco L, Keeffe JR, Khouri R, Olsen PC, Gazumyan A, Schaefer-Babajew D, Avila-Rios S, Nogueira L, Patel R, **Azzopardi SA**, Uhl LFK, Saeed M, Sevilla-Reyes EE, Agudelo M, Yao KH, Golijanin J, Gristick HB, Lee YE, Hurley A, Caskey M, Pai J, Oliveira T, Wunder EA Jr, Sacramento G, Nery N Jr, Orge C, Costa F, Reis MG, Thomas NM, Eisenreich T, Weinberger DM, de Almeida ARP, West AP Jr, Rice CM, Bjorkman PJ, Reyes-Teran G, Ko AI, MacDonald MR, Nussenzweig MC. Cell. 2017 May 4;169(4):597-609.e11. doi: 10.1016/j.cell.2017.04.024. PMID: 28475892; PMCID: PMC5492969.
*Expression of the receptor for hyaluronic acid mediated motility (RHAMM) is associated with poor prognosis and metastasis in non-small cell lung carcinoma.	Wang D, Narula N, **Azzopardi S**, Smith RS, Nasar A, Altorki NK, Mittal V, Somwar R, Stiles BM, Du YN. Oncotarget. 2016 Jun 28;7(26):39957-39969. doi: 10.18632/oncotarget.9554. PMID: 27220886; PMCID: PMC5129984.
**BABURYAN, SILVA**	
*The Srs2 helicase dampens DNA damage checkpoint by recycling RPA from chromatin.	Dhingra N, Kuppa S, Wei L, Pokhrel N, **Baburyan S**, Meng X, Antony E, Zhao X. Proc Natl Acad Sci U S A. 2021 Feb 23;118(8):e2020185118. doi: 10.1073/pnas.2020185118. PMID: 33602817; PMCID: PMC7923681.
**BOUMIZA, AIDA**	
*A unifying paradigm for transcriptional heterogeneity and squamous features in pancreatic ductal adenocarcinoma.	Hayashi A, Fan J, Chen R, Ho Y, Makohon-Moore AP, Lecomte N, Zhong Y, Hong J, Huang J, Sakamoto H, Attiyeh MA, Kohutek ZA, Zhang L, **Boumiza A**, Kappagantula R, Baez P, Bai J, Lisi M, Chadalavada K, Melchor JP, Wong W, Nanjangud GJ, Basturk O, O'Reilly EM, Klimstra DS, Hruban RH, Wood LD, Overholtzer M, Iacobuzio-Donahue CA. Nat Cancer. 2020 Jan;1(1):59–74. https://doi.org/10.1038/s43018-019-0010-1
**CASTILLO, RAYCHEL**	
*18F-FDG-PET/CT for systemic staging of patients with newly diagnosed ER-positive and HER2-positive breast cancer.	Ulaner GA, **Castillo R**, Wills J, Gönen M, Goldman DA. Eur J Nucl Med Mol Imaging. 2017 Aug;44(9):1420-1427. doi: 10.1007/s00259-017-3709-1. Epub 2017 Apr 29. PMID: 28456837.
*(18)F-FDG-PET/CT for systemic staging of newly diagnosed triple-negative breast cancer.	Ulaner GA, **Castillo R**, Goldman DA, Wills J, Riedl CC, Pinker-Domenig K, Jochelson MS, Gönen M. Eur J Nucl Med Mol Imaging. 2016 Oct;43(11):1937-44. doi: 10.1007/s00259-016-3402-9. Epub 2016 Apr 30. PMID: 27129866; PMCID: PMC5480318.
*Initial Results of a Prospective Clinical Trial of 18F-Fluciclovine PET/CT in Newly Diagnosed Invasive Ductal and Invasive Lobular Breast Cancers.	Ulaner GA, Goldman DA, Gönen M, Pham H, **Castillo R**, Lyashchenko SK, Lewis JS, Dang C. J Nucl Med. 2016 Sep;57(9):1350-6. doi: 10.2967/jnumed.115.170456. Epub 2016 Mar 3. PMID: 26940766.
**CHEN, CHRISTINE**	
Stereospecific Electrophilic Fluorination of Alkylcarbastannatrane Reagents.	Ma X, Diane M, Ralph G, **Chen C**, Biscoe MR. Angew Chem Int Ed Engl. 2017 Oct 2;56(41):12663-12667. doi: 10.1002/anie.201704672. Epub 2017 Sep 4. PMID: 28833888; PMCID: PMC8054978.
**CHOI, CRYSTAL**	
Optoacoustic Imaging of Glucagon-like Peptide-1 Receptor with a Near-Infrared Exendin-4 Analog.	Roberts S, Khera E, **Choi C**, Navaratna T, Grimm J, Thurber GM, Reiner T. J Nucl Med. 2021 Jun 1;62(6):839-848. doi: 10.2967/jnumed.120.252262. Epub 2020 Oct 23. PMID: 33097631.
*Acid specific dark quencher QC1 pHLIP for multi-spectral optoacoustic diagnoses of breast cancer.	Roberts S, Strome A, **Choi C**, Andreou C, Kossatz S, Brand C, Williams T, Bradbury M, Kircher MF, Reshetnyak YK, Grimm J, Lewis JS, Reiner T. Sci Rep. 2019 Jun 12;9(1):8550. doi: 10.1038/s41598-019-44873-1. PMID: 31189972; PMCID: PMC6561946.
*Sonophore-enhanced nanoemulsions for optoacoustic imaging of cancer.	Roberts S, Andreou C, **Choi C**, Donabedian P, Jayaraman M, Pratt EC, Tang J, Pérez-Medina C, Jason de la Cruz M, Mulder WJM, Grimm J, Kircher M, Reiner T. Chem Sci. 2018 May 18;9(25):5646-5657. doi: 10.1039/c8sc01706a. PMID: 30061998; PMCID: PMC6049522.
**DERDERIAN, CAMILLE**	
*Cancer cells deploy lipocalin-2 to collect limiting iron in leptomeningeal metastasis.	Chi Y, Remsik J, Kiseliovas V, **Derderian C**, Sener U, Alghader M, Saadeh F, Nikishina K, Bale T, Iacobuzio-Donahue C, Thomas T, Pe'er D, Mazutis L, Boire A. Science. 2020 Jul 17;369(6501):276-282. doi: 10.1126/science.aaz2193. PMID: 32675368; PMCID: PMC7816199.
*Leptomeningeal metastatic cells adopt two phenotypic states.	Remsik J, Chi Y, Tong X, Sener U, **Derderian C**, Park A, Saadeh F, Bale T, Boire A. Cancer Rep (Hoboken). 2020 Jan 29:e1236. doi: 10.1002/cnr2.1236. Epub ahead of print. PMID: 33372403; PMCID: PMC7772527.
**ELEY, ELEANOR**	
*Developing a MLC modifier program to improve fiducial detection for MV/kV imaging during hypofractionated prostate volumetric modulated arc therapy.	Happersett L, Wang P, Zhang P, Mechalakos J, Li G, **Eley E**, Zelefsky M, Mageras G, Damato AL, Hunt M. J Appl Clin Med Phys. 2019 Jun;20(6):120-124. doi: 10.1002/acm2.12614. Epub 2019 May 22. PMID: 31116478; PMCID: PMC6560246.
**FARUQUE, PROMIE**	
*T follicular helper phenotype predicts response to histone deacetylase inhibitors in relapsed/refractory peripheral T-cell lymphoma.	Ghione P, **Faruque P**, Mehta-Shah N, Seshan V, Ozkaya N, Bhaskar S, Yeung J, Spinner MA, Lunning M, Inghirami G, Moskowitz A, Galasso N, Ganesan N, van der Weyden C, Ruan J, Prince HM, Trotman J, Advani R, Dogan A, Horwitz S. Blood Adv. 2020 Oct 13;4(19):4640-4647. doi: 10.1182/bloodadvances.2020002396. PMID: 33002132; PMCID: PMC7556143.
*Phospholipase D-dependent mTOR complex 1 (mTORC1) activation by glutamine.	Bernfeld E, Menon D, Vaghela V, Zerin I, **Faruque P**, Frias MA, Foster DA. J Biol Chem. 2018 Oct 19;293(42):16390-16401. doi: 10.1074/jbc.RA118.004972. Epub 2018 Sep 7. PMID: 30194281; PMCID: PMC6200938.
**GOPAL, ARIANA**	
The Functionality, Evidence, and Privacy Issues Around Smartphone Apps for the Top Neuropsychiatric Conditions.	Minen MT, **Gopal A**, Sahyoun G, Stieglitz E, Torous J. J Neuropsychiatry Clin Neurosci. 2021 Winter;33(1):72-79. doi: 10.1176/appi.neuropsych.19120353. Epub 2020 Jul 16. PMID: 32669020; PMCID: PMC8670295.

Cis-regulatory analysis of Onecut1 expression in fate-restricted retinal progenitor cells.	Patoori S, Jean-Charles N, **Gopal A**, Sulaiman S, Gopal S, Wang B, Souferi B, Emerson MM. Neural Dev. 2020 Mar 19;15(1):5. doi: 10.1186/s13064-020-00142-w. PMID: 32192535; PMCID: PMC7082998.
A Pilot Randomized Controlled Trial to Assess the Impact of Motivational Interviewing on Initiating Behavioral Therapy for Migraine. Headache.	Minen MT, Sahyoun G, **Gopal A**, Levitan V, Pirraglia E, Simon NM, Halpern A. 2020 Feb;60(2):441-456. doi: 10.1111/head.13738. Epub 2020 Jan 24. PMID: 31981227; PMCID: PMC7754247.
**GUBER, DAVID**	
*Synthesis of an Alkynyl Methylglyoxal Probe to Investigate Nonenzymatic Histone Glycation.	Zheng Q, Maksimovic I, Upad A, **Guber D**, David Y. J Org Chem. 2020 Feb 7;85(3):1691-1697. doi: 10.1021/acs.joc.9b02504. Epub 2020 Jan 7. PMID: 31875401; PMCID: PMC8018604.
**GURU, NAVJOT**	
*[18F]PARPi Imaging Is Not Affected by HPV Status In Vitro.	**Guru N**, Demétrio De Souza França P, Pirovano G, Huang C, Patel SG, Reiner T. Mol Imaging. 2021 Jan 20;2021:6641397. doi: 10.1155/2021/6641397. PMID: 34194286; PMCID: PMC8205605.
*A phase I study of a PARP1-targeted topical fluorophore for the detection of oral cancer.	Demétrio de Souza França P, Kossatz S, Brand C, Karassawa Zanoni D, Roberts S, **Guru N**, Adilbay D, Mauguen A, Valero Mayor C, Weber WA, Schöder H, Ghossein RA, Ganly I, Patel SG, Reiner T. Eur J Nucl Med Mol Imaging. 2021 Oct;48(11):3618-3630. doi: 10.1007/s00259-021-05372-6. Epub 2021 May 5. PMID: 33954826.
*PARP-Targeted Auger Therapy in p53 Mutant Colon Cancer Xenograft Mouse Models.	Wilson T, Pirovano G, Xiao G, Samuels Z, Roberts S, Viray T, **Guru N**, Zanzonico P, Gollub M, Pillarsetty NVK, Reiner T, Bargonetti J. Mol Pharm. 2021 Sep 6;18(9):3418-3428. doi: 10.1021/acs.molpharmaceut.1c00323. Epub 2021 Jul 28. PMID: 34318678; PMCID: PMC8686831.
*PARP1: A Potential Molecular Marker to Identify Cancer During Colposcopy Procedures.	de Souza França PD, **Guru N**, Kostolansky AR, Mauguen A, Pirovano G, Kossatz S, Roberts S, Abrahão M, Patel SG, Park KJ, Reiner T, Jewell E. J Nucl Med. 2021 Jul 1;62(7):941-948. doi: 10.2967/jnumed.120.253575. Epub 2020 Nov 13. PMID: 33188153.
*Bimodal Imaging of Mouse Peripheral Nerves with Chlorin Tracers.	Gonzales J, Hernández-Gil J, Wilson TC, Adilbay D, Cornejo M, Demétrio de Souza Franca P, **Guru N**, Schroeder CI, King GF, Lewis JS, Reiner T. Mol Pharm. 2021 Mar 1;18(3):940-951. doi: 10.1021/acs.molpharmaceut.0c00946. Epub 2021 Jan 6. PMID: 33404254; PMCID: PMC7920913.
*Fluorescence-guided resection of tumors in mouse models of oral cancer.	Demétrio de Souza França P, **Guru N**, Roberts S, Kossatz S, Mason C, Abrahão M, Ghossein RA, Patel SG, Reiner T. Sci Rep. 2020 Jul 7;10(1):11175. doi: 10.1038/s41598-020-67958-8. PMID: 32636416; PMCID: PMC7341853.
*Improved radiosynthesis of 123I-MAPi, an auger theranostic agent.	Wilson TC, Jannetti SA, **Guru N**, Pillarsetty N, Reiner T, Pirovano G. Int J Radiat Biol. 2020 Jul 2:1-7. doi: 10.1080/09553002.2020.1781283. Epub ahead of print. PMID: 32552309; PMCID: PMC7775866.
*Targeted Brain Tumor Radiotherapy Using an Auger Emitter.	Pirovano G, Jannetti SA, Carter LM, Sadique A, Kossatz S, **Guru N**, Demétrio De Souza França P, Maeda M, Zeglis BM, Lewis JS, Humm JL, Reiner T. Clin Cancer Res. 2020 Jun 15;26(12):2871-2881. doi: 10.1158/1078-0432.CCR-19-2440. Epub 2020 Feb 17. PMID: 32066626; PMCID: PMC7299758.
*Fluorine-18 labeled poly (ADP-ribose) polymerase1 inhibitor as a potential alternative to 2-deoxy-2-[18F]fluoro-d-glucose positron emission tomography in oral cancer imaging.	Demétrio de Souza França P, Roberts S, Kossatz S, **Guru N**, Mason C, Zanoni DK, Abrahão M, Schöder H, Ganly I, Patel SG, Reiner T. Nucl Med Biol. 2020 May-Jun;84-85:80-87. doi: 10.1016/j.nucmedbio.2020.01.004. Epub 2020 Jan 23. PMID: 32135475; PMCID: PMC7253343.
*Fluorescence labeling of a NaV1.7-targeted peptide for near-infrared nerve visualization.	Gonzales J, Pirovano G, Chow CY, de Souza Franca PD, Carter LM, Klint JK, **Guru N**, Lewis JS, King GF, Reiner T. EJNMMI Res. 2020 May 14;10(1):49. doi: 10.1186/s13550-020-00630-4. PMID: 32409881; PMCID: PMC7225226.
*An 89Zr-HDL PET Tracer Monitors Response to a CSF1R Inhibitor.	Mason CA, Kossatz S, Carter LM, Pirovano G, Brand C, **Guru N**, Pérez-Medina C, Lewis JS, Mulder WJM, Reiner T. J Nucl Med. 2020 Mar;61(3):433-436. doi: 10.2967/jnumed.119.230466. Epub 2019 Aug 16. PMID: 31420495; PMCID: PMC7067531.
*Fluorescence Imaging of Peripheral Nerves by a Nav1.7-Targeted Inhibitor Cystine Knot Peptide.	Gonzales J, Demetrio de Souza Franca P, Jiang Y, Pirovano G, Kossatz S, **Guru N**, Yarilin D, Agwa AJ, Schroeder CI, Patel SG, Ganly I, King GF, Reiner T. Bioconjug Chem. 2019 Nov 20;30(11):2879-2888. doi: 10.1021/acs.bioconjchem.9b00612. Epub 2019 Nov 8. PMID: 31647222; PMCID: PMC7372312.
**HAQUE, NOSHIN**	
*Oncology Care Provider Training in Empathic Communication Skills to Reduce Lung Cancer Stigma.	Banerjee SC, **Haque N**, Schofield EA, Williamson TJ, Martin CM, Bylund CL, Shen MJ, Rigney M, Hamann HA, Parker PA, McFarland DC, Park BJ, Molena D, Moreno A, Ostroff JS. Chest. 2021 May;159(5):2040-2049. doi: 10.1016/j.chest.2020.11.024. Epub 2020 Dec 16. PMID: 33338443; PMCID: PMC8129726.
*Responding empathically to patients: a communication skills training module to reduce lung cancer stigma.	Banerjee SC, **Haque N**, Bylund CL, Shen MJ, Rigney M, Hamann HA, Parker PA, Ostroff JS. Transl Behav Med. 2021 Mar 16;11(2):613-618. doi: 10.1093/tbm/ibaa011. PMID: 32080736; PMCID: PMC7963287.
*Dispositional shame and guilt as predictors of depressive symptoms and anxiety among adults with lung cancer: The mediational role of internalized stigma.	Williamson TJ, Ostroff JS, **Haque N**, Martin CM, Hamann HA, Banerjee SC, Shen MJ. Stigma Health. 2020 Nov;5(4):425-433. doi: 10.1037/sah0000214. Epub 2020 Jan 16. PMID: 34027060; PMCID: PMC8132596.
Smoking policies in the home have less influence on cigarettes per day and nicotine dependence level among African American than White smokers: A cross-sectional analysis.	Ruglass LM, Root JC, Dambreville N, Shevorykin A, **Haque N**, Sun V, Sheffer CE, Melara RD. J Natl Med Assoc. 2019 Dec;111(6):606-615. doi: 10.1016/j.jnma.2019.07.002. Epub 2019 Jul 30. PMID: 31375277; PMCID: PMC6925645.
*Understanding cognitive and emotional illness representations of South Asian head and neck cancer survivors: a qualitative study.	Banerjee SC, Camacho-Rivera M, **Haque N**, Flynn L, Thomas J, Smith P, Sheffer C, Ostroff JS. Ethn Health. 2019 Aug 26:1-18. doi: 10.1080/13557858.2019.1625872. Epub ahead of print. PMID: 31448959; PMCID: PMC7524586.
*Structured Analysis of Empathic Opportunities and Physician Responses during Lung Cancer Patient-Physician Consultations.	Johnson Shen M, Ostroff JS, Hamann HA, **Haque N**, Banerjee SC, McFarland DC, Molena D, Bylund CL. J Health Commun. 2019;24(9):711-718. doi: 10.1080/10810730.2019.1665757. Epub 2019 Sep 16. PMID: 31525115; PMCID: PMC6884685.
**HAYNES, ASHLEY**	
*Excess TGF-β mediates muscle weakness associated with bone metastases in mice.	Waning DL, Mohammad KS, Reiken S, Xie W, Andersson DC, John S, Chiechi A, Wright LE, Umanskaya A, Niewolna M, Trivedi T, Charkhzarrin S, Khatiwada P, Wronska A, **Haynes A**, Benassi MS, Witzmann FA, Zhen G, Wang X, Cao X, Roodman GD, Marks AR, Guise TA. Nat Med. 2015 Nov;21(11):1262-1271. doi: 10.1038/nm.3961. Epub 2015 Oct 12. PMID: 26457758; PMCID: PMC4636436.
**HONG, SEONG IM**	
*Association between morphologic CT imaging traits and prognostically relevant gene signatures in women with high-grade serous ovarian cancer: a hypothesis-generating study.	Vargas HA, Miccò M, **Hong SI**, Goldman DA, Dao F, Weigelt B, Soslow RA, Hricak H, Levine DA, Sala E. Radiology. 2015 Mar;274(3):742-51. doi: 10.1148/radiol.14141477. Epub 2014 Nov 10. PMID: 25383459; PMCID: PMC4455661.
Characterization of a subpopulation of developing cortical interneurons from human iPSCs within serum-free embryoid bodies.	Nestor MW, Jacob S, Sun B, Prè D, Sproul AA, **Hong SI**, Woodard C, Zimmer M, Chinchalongporn V, Arancio O, Noggle SA. Am J Physiol Cell Physiol. 2015 Feb 1;308(3):C209-19. doi: 10.1152/ajpcell.00263.2014. Epub 2014 Nov 12. PMID: 25394470; PMCID: PMC4312839.

**HUANG, CIEN**	
*Sensors and Inhibitors for the Detection of Ataxia Telangiectasia Mutated (ATM) Protein Kinase.	**Huang C**, Filippone NR, Reiner T, Roberts S. Mol Pharm. 2021 Jul 5;18(7):2470-2481. doi: 10.1021/acs.molpharmaceut.1c00166. Epub 2021 Jun 14. PMID: 34125542.
**[18F]PARPi Imaging Is Not Affected by HPV Status In Vitro.*	*Guru N, Demétrio De Souza França P, Pirovano G, **Huang C**, Patel SG, Reiner T. Mol Imaging. 2021 Jan 20;2021:6641397. doi: 10.1155/2021/6641397. PMID: 34194286; PMCID: PMC8205605.*
**IYER, SAIPRIYA**	
*Minimal disseminated disease evaluation and outcome in trilateral retinoblastoma.	Torbidoni AV, Sampor C, Laurent VE, Aschero R, **Iyer S**, Rossi J, Alderete D, Alonso DF, Szijan I, Chantada GL. Br J Ophthalmol. 2018 Nov;102(11):1597-1601. doi: 10.1136/bjophthalmol-2018-312263. Epub 2018 Aug 27. PMID: 30150278.
*Retinoblastoma Vitreous Seed Clouds (Class 3): A Comparison of Treatment with Ophthalmic Artery Chemosurgery with or without Intravitreous and Periocular Chemotherapy.	Francis JH, **Iyer S**, Gobin YP, Brodie SE, Abramson DH. Ophthalmology. 2017 Oct;124(10):1548-1555. doi: 10.1016/j.ophtha.2017.04.010. Epub 2017 May 22. PMID: 28545735.
*Growth of Uveal Melanoma following Intravitreal Bevacizumab.	Francis JH, Kim J, Lin A, Folberg R, **Iyer S**, Abramson DH. Ocul Oncol Pathol. 2017 Jul;3(2):117-121. doi: 10.1159/000450859. Epub 2016 Nov 12. PMID: 28868282; PMCID: PMC5566762.
*Intraocular Pressure Changes Following Intravitreal Melphalan and Topotecan for the Treatment of Retinoblastoma With Vitreous Seeding.	Karl MD, Francis JH, **Iyer S**, Marr B, Abramson DH. J Pediatr Ophthalmol Strabismus. 2017 May 1;54(3):185-190. doi: 10.3928/01913913-20161116-01. Epub 2017 Jan 17. PMID: 28092395; PMCID: PMC5473509.
**IZQUIERDO, KAREN**	
*A Systematic Review of the Impact of Patient-Physician Non-English Language Concordance on Quality of Care and Outcomes.	Diamond L, **Izquierdo K**, Canfield D, Matsoukas K, Gany F. J Gen Intern Med. 2019 Aug;34(8):1591-1606. doi: 10.1007/s11606-019-04847-5. Epub 2019 May 30. PMID: 31147980; PMCID: PMC6667611.
**JAFFERY, SANA**	
*Carotid sparing intensity-modulated radiation therapy achieves comparable locoregional control to conventional radiotherapy in T1-2N0 laryngeal carcinoma.	Zumsteg ZS, Riaz N, **Jaffery S**, Hu M, Gelblum D, Zhou Y, Mychalczak B, Zelefsky MJ, Wolden S, Rao S, Lee NY. Oral Oncol. 2015 Jul;51(7):716-23. doi: 10.1016/j.oraloncology.2015.02.003. Epub 2015 May 7. PMID: 25958831; PMCID: PMC5508531.
**JUAREZ, JESSICA**	
*18F-FDG PET/CT for Systemic Staging of Newly Diagnosed Breast Cancer in Men.	Ulaner GA, **Juarez J**, Riedl CC, Goldman DA. J Nucl Med. 2019 Apr;60(4):472-477. doi: 10.2967/jnumed.118.217836. Epub 2018 Sep 20. PMID: 30237211; PMCID: PMC6448458.
**KAZI, ANAN**	
Herpes-zoster associated urinary retention in a 57-year-old immunocompromised male.	Au VH, **Kazi A**, Bruha M, Weiss J. Urol Case Rep. 2020 Nov 19;34:101498. doi: 10.1016/j.eucr.2020.101498. PMID: 33294380; PMCID: PMC7691546.
**KOGAN, DIANE**	
*5-Aminoimidazole-4-carboxamide-1-β-4-ribofuranoside (AICAR) enhances the efficacy of rapamycin in human cancer cells.	Mukhopadhyay S, Chatterjee A, **Kogan D**, Patel D, Foster DA. Cell Cycle. 2015;14(20):3331-9. doi: 10.1080/15384101.2015.1087623. PMID: 26323019; PMCID: PMC4825547.
**KWONG, ERICA**	
*Peripheral blood clinical laboratory variables associated with outcomes following combination nivolumab and ipilimumab immunotherapy in melanoma.	Rosner S, **Kwong E**, Shoushtari AN, Friedman CF, Betof AS, Brady MS, Coit DG, Callahan MK, Wolchok JD, Chapman PB, Panageas KS, Postow MA. Cancer Med. 2018 Mar;7(3):690-697. doi: 10.1002/cam4.1356. Epub 2018 Feb 22. PMID: 29468834; PMCID: PMC5852343.
**LEEOLOU, MELISSA**	
Inhibiting Fibroblast Mechanotransduction Modulates Severity of Idiopathic Pulmonary Fibrosis.	Trotsyuk AA, Chen K, Hyung S, Ma KC, Henn D, Mermin-Bunnell AM, Mittal S, Padmanabhan J, Larson MR, Steele SR, Sivaraj D, Bonham CA, Noishiki C, Rodrigues M, Jiang Y, Jing S, Niu S, Chattopadhyay A, Perrault DP, **Leeolou MC**, Fischer KS, Gurusankar G, Kussie HC, Wan DC, Januszyk M, Longaker MT, Gurtner GC. Adv Wound Care (New Rochelle). 2021 Nov 30. doi: 10.1089/wound.2021.0077. Epub ahead of print. PMID: 34544267.
**LUNG, BETTY**	
*Complete Responses to Mitotane in Metastatic Adrenocortical Carcinoma-A New Look at an Old Drug.	Reidy-Lagunes DL, **Lung B**, Untch BR, Raj N, Hrabovsky A, Kelly C, Gerst S, Katz S, Kampel L, Chou J, Gopalan A, Saltz LB. Oncologist. 2017 Sep;22(9):1102-1106. doi: 10.1634/theoncologist.2016-0459. Epub 2017 May 30. PMID: 28559412; PMCID: PMC5599197.
*Continuous Trastuzumab Therapy in Breast Cancer Patients With Asymptomatic Left Ventricular Dysfunction.	Yu AF, Yadav NU, Eaton AA, **Lung BY**, Thaler HT, Liu JE, Hudis CA, Dang CT, Steingart RM. Oncologist. 2015 Oct;20(10):1105-10. doi: 10.1634/theoncologist.2015-0125. Epub 2015 Aug 3. PMID: 26240135; PMCID: PMC4591940.
*Trastuzumab interruption and treatment-induced cardiotoxicity in early HER2-positive breast cancer.	Yu AF, Yadav NU, **Lung BY**, Eaton AA, Thaler HT, Hudis CA, Dang CT, Steingart RM. Breast Cancer Res Treat. 2015 Jan;149(2):489-95. doi: 10.1007/s10549-014-3253-7. Epub 2015 Jan 1. PMID: 25552363; PMCID: PMC4970316.
**MAINARICH, SHIANA**	
*Survival Prediction in Pancreatic Ductal Adenocarcinoma by Quantitative Computed Tomography Image Analysis.	Attiyeh MA, Chakraborty J, Doussot A, Langdon-Embry L, **Mainarich S**, Gönen M, Balachandran VP, D'Angelica MI, DeMatteo RP, Jarnagin WR, Kingham TP, Allen PJ, Simpson AL, Do RK. Ann Surg Oncol. 2018 Apr;25(4):1034-1042. doi: 10.1245/s10434-017-6323-3. Epub 2018 Jan 29. PMID: 29380093; PMCID: PMC6752719.
**MAKURUMIDZE, GETRUDE**	
CCR10 expression is required for the adjuvant activity of the mucosal chemokine CCL28 when delivered in the context of an HIV-1 Env DNA vaccine.	Gary EN, Kathuria N, **Makurumidze G**, Curatola A, Ramamurthi A, Bernui ME, Myles D, Yan J, Pankhong P, Muthumani K, Haddad E, Humeau L, Weiner DB, Kutzler MA. Vaccine. 2020 Mar 4;38(11):2626-2635. doi: 10.1016/j.vaccine.2020.01.023. Epub 2020 Feb 10. PMID: 32057572.
HIV/AIDS psychiatry - a paradigm for HIV prevention and integrated compassionate care.	Cohen MAA, **Makurumidze G**, Pereira LF, Bourgeois JA, Cozza KL. World Psychiatry. 2019 Jun;18(2):240-241. doi: 10.1002/wps.20643. PMID: 31059622; PMCID: PMC6502402.
**MARKOVA, SVETLANA**	
*Evaluation of automatic contour propagation in T2-weighted 4DMRI for normal-tissue motion assessment using internal organ-at-risk volume (IRV).	Zhang J, **Markova S**, Garcia A, Huang K, Nie X, Choi W, Lu W, Wu A, Rimner A, Li G. J Appl Clin Med Phys. 2018 Sep;19(5):598-608. doi: 10.1002/acm2.12431. Epub 2018 Aug 15. PMID: 30112797; PMCID: PMC6123161.

*Novel Super-Resolution Approach to Time-Resolved Volumetric 4-Dimensional Magnetic Resonance Imaging With High Spatiotemporal Resolution for Multi-Breathing Cycle Motion Assessment.	Li G, Wei J, Kadbi M, Moody J, Sun A, Zhang S, **Markova S**, Zakian K, Hunt M, Deasy JO. Int J Radiat Oncol Biol Phys. 2017 Jun 1;98(2):454-462. doi: 10.1016/j.ijrobp.2017.02.016. Epub 2017 Feb 17. PMID: 28463165; PMCID: PMC5481849.
**MCILVRIDE, ALLISON**	
*Prospective Analysis Using a Novel CNN Algorithm to Distinguish Atypical Ductal Hyperplasia From Ductal Carcinoma in Situ in Breast.	Mutasa S, Chang P, Nemer J, Van Sant EP, Sun M, **McIlvride A**, Siddique M, Ha R. Clin Breast Cancer. 2020 Dec;20(6):e757-e760. doi: 10.1016/j.clbc.2020.06.001. Epub 2020 Jun 7. PMID: 32680766; PMCID: PMC8207833.
Development of a New Patient-reported Outcome Instrument to Evaluate Treatments for Scars: The SCAR-Q.	Klassen AF, Ziolkowski N, Mundy LR, Miller HC, **McIlvride A**, DiLaura A, Fish J, Pusic AL. Plast Reconstr Surg Glob Open. 2018 Apr 24;6(4):e1672. doi: 10.1097/GOX.0000000000001672. PMID: 2987Klassen AF, Ziolkowski N, Mundy LR, Miller HC, McIlvride A, DiLaura A, Fish J, Pusic AL. Plast Reconstr Surg Glob Open. 2018 Apr 24;6(4):e1672. doi: 10.1097/GOX.0000000000001672. PMID: 29876160; PMCID: PMC5977950.6160; PMCID: PMC5977950.
**MCINTYRE, DANA**	
Brands with personalities - good for businesses, but bad for public health? A content analysis of how food and beverage brands personify themselves on Twitter.	Greene T, Seet C, Rodríguez Barrio A, **McIntyre D**, Kelly B, Bragg MA. Public Health Nutr. 2021 Apr 6:1-10. doi: 10.1017/S1368980021001439. Epub ahead of print. PMID: 33820575.
**MCMILLAN, JULIA**	
*Comparing the reporting and conduct quality of exercise and pharmacological randomised controlled trials: a systematic review.	Adams SC, **McMillan J**, Salline K, Lavery J, Moskowitz CS, Matsoukas K, Chen MMZ, Santa Mina D, Scott JM, Jones LW. BMJ Open. 2021 Aug 11;11(8):e048218. doi: 10.1136/bmjopen-2020-048218. PMID: 34380726; PMCID: PMC8359527.
**MEHTA, KRISHA**	
*Regulation of the error-prone DNA polymerase Polκ by oncogenic signaling and its contribution to drug resistance.	Temprine K, Campbell NR, Huang R, Langdon EM, Simon-Vermot T, **Mehta K**, Clapp A, Chipman M, White RM. Sci Signal. 2020 Apr 28;13(629):eaau1453. doi: 10.1126/scisignal.aau1453. PMID: 32345725; PMCID: PMC7428051.
**MIKHAYLOV, DANIELA**	
Transcriptomic Profiling of Tape-Strips From Moderate to Severe Atopic Dermatitis Patients Treated With Dupilumab.	**Mikhaylov D**, Del Duca E, Olesen CM, He H, Wu J, Ungar B, Estrada Y, Zhang N, Chowdhury M, Clausen ML, Krueger JG, Pavel AB, Agner T, Guttman-Yassky E. Dermatitis. 2021 Oct 1;32(1S):S71-S80. doi: 10.1097/DER.0000000000000764. PMID: 34405829.
Proteomic signatures of inflammatory skin diseases: a focus on atopic dermatitis.	**Mikhaylov D**, Del Duca E, Guttman-Yassky E. Expert Rev Proteomics. 2021 May;18(5):345-361. doi: 10.1080/14789450.2021.1935247. Epub 2021 Jun 4. PMID: 34033497.
Systemic Psoriasis Therapies and Comorbid Disease in Patients with Psoriasis: A Review of Potential Risks and Benefits.	**Mikhaylov D**, Hashim PW, Nektalova T, Goldenberg G. J Clin Aesthet Dermatol. 2019 Jun;12(6):46-54. Epub 2019 Jun 1. PMID: 31360288; PMCID: PMC6624011.
A randomized placebo-controlled single-center pilot study of the safety and efficacy of apremilast in subjects with moderate-to-severe alopecia areata.	**Mikhaylov D**, Pavel A, Yao C, Kimmel G, Nia J, Hashim P, Vekaria AS, Taliercio M, Singer G, Karalekas R, Baum D, Mansouri Y, Lebwohl MG, Guttman-Yassky E. Arch Dermatol Res. 2019 Jan;311(1):29-36. doi: 10.1007/s00403-018-1876-y. Epub 2018 Nov 11. PMID: 30417279.
A Sleep Hygiene Intervention to Improve Sleep Quality for Hospitalized Patients.	Herscher M, **Mikhaylov D**, Barazani S, Sastow D, Yeo I, Dunn AS, Cho HJ. Jt Comm J Qual Patient Saf. 2021 Jun;47(6):343-346. doi: 10.1016/j.jcjq.2021.02.003. Epub 2021 Feb 11. PMID: 33744173.
Tape-strips provide a minimally invasive approach to track therapeutic response to topical corticosteroids in atopic dermatitis patients.	Olesen CM, Pavel AB, Wu J, **Mikhaylov D**, Del Duca E, Estrada Y, Krueger JG, Zhang N, Clausen ML, Agner T, Guttman-Yassky E. J Allergy Clin Immunol Pract. 2021 Jan;9(1):576-579.e3. doi: 10.1016/j.jaip.2020.08.037. Epub 2020 Sep 2. PMID: 32889222.
Moisturizers: A Comparison Based on Allergens and Economic Value.	Chou M, **Mikhaylov D**, Lazic Strugar T. Dermatitis. 2018 Nov/Dec;29(6):339-344. doi: 10.1097/DER.0000000000000413. PMID: 30346325.
**MILLAN, MICAELA**	
*Multi-sample measurement of hyperpolarized pyruvate-to-lactate flux in melanoma cells.	Lees H, **Millan M**, Ahamed F, Eskandari R, Granlund KL, Jeong S, Keshari KR. NMR Biomed. 2021 Mar;34(3):e4447. doi: 10.1002/nbm.4447. Epub 2020 Dec 12. PMID: 33314422; PMCID: PMC8288443.
**NAVITSKI, ANASTASIA**	
*Baseline risk of hematologic malignancy at initiation of frontline PARP inhibitor maintenance for BRCA1/2-associated ovarian cancer.	**Navitski A**, Al-Rawi DH, Liu Y, Rubinstein MM, Friedman CF, Rampal RK, Mandelker DL, Cadoo K, O'Cearbhaill RE. Gynecol Oncol Rep. 2021 Oct 5;38:100873. doi: 10.1016/j.gore.2021.100873. PMID: 34926756; PMCID: PMC8651772.
**Cohesin Members Stag1 and Stag2 Display Distinct Roles in Chromatin Accessibility and Topological Control of HSC Self-Renewal and Differentiation.*	*Viny AD, Bowman RL, Liu Y, Lavallée VP, Eisman SE, Xiao W, Durham BH, **Navitski A**, Park J, Braunstein S, Alija B, Karzai A, Csete IS, Witkin M, Azizi E, Baslan T, Ott CJ, Pe'er D, Dekker J, Koche R, Levine RL. Cell Stem Cell. 2019 Nov 7;25(5):682-696.e8. doi: 10.1016/j.stem.2019.08.003. Epub 2019 Sep 5. PMID: 31495782; PMCID: PMC6842438.*
**NGUY, SUSANNA**	
*Outcomes of HER2-positive non-metastatic breast cancer patients treated with anti-HER2 therapy without chemotherapy.	**Nguy S**, Wu SP, Oh C, Gerber NK. Breast Cancer Res Treat. 2021 Jun;187(3):815-830. doi: 10.1007/s10549-021-06115-9. Epub 2021 Feb 15. PMID: 33590386.
*Radiotherapy in Metastatic Oropharyngeal Cancer.	**Nguy S**, Oh C, Karp JM, Wu SP, Li Z, Persky MJ, Hu KS, Givi B, Tam MM. Laryngoscope. 2021 Jun;131(6):E1847-E1853. doi: 10.1002/lary.29245. Epub 2020 Nov 3. PMID: 33141455.
*PD-L1 engagement on T cells promotes self-tolerance and suppression of neighboring macrophages and effector T cells in cancer.	Diskin B, Adam S, Cassini MF, Sanchez G, Liria M, Aykut B, Buttar C, Li E, Sundberg B, Salas RD, Chen R, Wang J, Kim M, Farooq MS, **Nguy S**, Fedele C, Tang KH, Chen T, Wang W, Hundeyin M, Rossi JAK, Kurz E, Haq MIU, Karlen J, Kruger E, Sekendiz Z, Wu D, Shadaloey SAA, Baptiste G, Werba G, Selvaraj S, Loomis C, Wong KK, Leinwand J, Miller G. Nat Immunol. 2020 Apr;21(4):442-454. doi: 10.1038/s41590-020-0620-x. Epub 2020 Mar 9. PMID: 32152508.
*Radiation Therapy Induces Macrophages to Suppress T-Cell Responses Against Pancreatic Tumors in Mice.	Seifert L, Werba G, Tiwari S, Giao Ly NN, **Nguy S**, Alothman S, Alqunaibit D, Avanzi A, Daley D, Barilla R, Tippens D, Torres-Hernandez A, Hundeyin M, Mani VR, Hajdu C, Pellicciotta I, Oh P, Du K, Miller G. Gastroenterology. 2016 Jun;150(7):1659-1672.e5. doi: 10.1053/j.gastro.2016.02.070. Epub 2016 Mar 3. PMID: 26946344; PMCID: PMC4909514.
*TLR9 ligation in pancreatic stellate cells promotes tumorigenesis.	Zambirinis CP, Levie E, **Nguy S**, Avanzi A, Barilla R, Xu Y, Seifert L, Daley D, Greco SH, Deutsch M, Jonnadula S, Torres-Hernandez A, Tippens D, Pushalkar S, Eisenthal A, Saxena D, Ahn J, Hajdu C, Engle DD, Tuveson D, Miller G. J Exp Med. 2015 Nov 16;212(12):2077-94. doi: 10.1084/jem.20142162. Epub 2015 Oct 19. PMID: 26481685; PMCID: PMC4647258.
*Toxicity and disease-related outcomes after radiotherapy for head and neck cancer in human immunodeficiency virus-positive patients.	Grew DJ, Cooper BT, **Nguy S**, Halperin J, Sanfilippo NJ. Front Oncol. 2014 Nov 10;4:316. doi: 10.3389/fonc.2014.00316. PMID: 25426448; PMCID: PMC4226241.

A Western-fed diet increases plasma HDL and LDL-cholesterol levels in apoD-/- mice.	Ali K, Abo-Ali EM, Kabir MD, Riggins B, **Nguy S**, Li L, Srivastava U, Thinn SM. PLoS One. 2014 Dec 30;9(12):e115744. doi: 10.1371/journal.pone.0115744. PMID: 25548917; PMCID: PMC4280175.
**NIKISHINA, KATIE**	
*Cancer cells deploy lipocalin-2 to collect limiting iron in leptomeningeal metastasis.	Chi Y, Remsik J, Kiseliovas V, Derderian C, Sener U, Alghader M, Saadeh F, **Nikishina K**, Bale T, Iacobuzio-Donahue C, Thomas T, Pe'er D, Mazutis L, Boire A. Science. 2020 Jul 17;369(6501):276-282. doi: 10.1126/science.aaz2193. PMID: 32675368; PMCID: PMC7816199.
**NOEL, JOVANKA**	
*Liposomal Bupivacaine Versus Bupivacaine Hydrochloride for Intercostal Nerve Blockade in Minimally Invasive Thoracic Surgery.	Pedoto A, **Noel J**, Park BJ, Amar D. J Cardiothorac Vasc Anesth. 2021 May;35(5):1393-1398. doi: 10.1053/j.jvca.2020.11.067. Epub 2020 Dec 2. PMID: 33376072.
**NWORA, EMANUELA**	
*Assessing Cerebrospinal Fluid Flow Dynamics in Pediatric Patients with Central Nervous System Tumors Treated with Intraventricular Radioimmunotherapy.	Kramer K, Donzelli M, **Nwora E**, Pandit-Taskar N.J Nucl Med. 2020 May;61(5):662-664. doi: 10.2967/jnumed.119.232678. Epub 2020 Jan 31. PMID: 32005772; PMCID: PMC7198389.
**OH, CHRISTIANA (CHO ROK)**	
Tunable optical metamaterial-based sensors enabled by closed bipolar electrochemistry.	Crouch GM, **Oh C**, Fu K, Bohn PW. Analyst. 2019 Nov 7;144(21):6240-6246. doi: 10.1039/c9an01137d. Epub 2019 Sep 20. PMID: 31538160; PMCID: PMC8030654.
**PALMA, LAURA**	
Hox gene expression determines cell fate of adult periosteal stem/progenitor cells.	Bradaschia-Correa V, Leclerc K, Josephson AM, Lee S, **Palma L**, Litwa HP, Neibart SS, Huo JC, Leucht P. Sci Rep. 2019 Mar 25;9(1):5043. doi: 10.1038/s41598-019-41639-7. Erratum in: Sci Rep. 2020 Feb 18;10(1):3220. PMID: 30911091; PMCID: PMC6434021.
Author Correction: Hox gene expression determines cell fate of adult periosteal stem/progenitor cells.	*Bradaschia-Correa V, Leclerc K, Josephson AM, Lee S,* ***Palma L*** *, Litwa HP, Neibart SS, Huo JC, Leucht P. Sci Rep. 2020 Feb 18;10(1):3220. doi: 10.1038/s41598-020-59764-z. Erratum for: Sci Rep. 2019 Mar 25;9(1):5043. PMID: 32066822; PMCID: PMC7026393.*
**PEÑA, DARWIN**	
Apolipoproteins L1-6 share key cation channel-regulating residues but have different membrane insertion and ion conductance properties.	Pant J, Giovinazzo JA, Tuka LS, **Peña D**, Raper J, Thomson R. J Biol Chem. 2021 Aug;297(2):100951. doi: 10.1016/j.jbc.2021.100951. Epub 2021 Jul 10. PMID: 34252458; PMCID: PMC8358165.
**PETERS, ANISIA**	
A new approach for inferring traffic-related air pollution: Use of radar-calibrated crowd-sourced traffic data	Hilpert M, Johnson M, Kioumourtzoglou MA, Domingo-Relloso A, **Peters A**, Adria-Mora B, Hernández D, Ross J, Chillrud SN. Environ Int. 2019 Jun;127:142-159. doi: 10.1016/j.envint.2019.03.026. Epub 2019 Mar 23. PMID: 30913459; PMCID: PMC7013362.
**PIRACHA, YUMNA**	
A Novel Approach to Fracture Resistance Using Horizontal Posts after Endodontic Therapy: A Case Report and Review of Literature.	Kim SG, Kim SS, Levine JL, **Piracha YS**, Solomon CS.J Endod. 2020 Apr;46(4):545-550. doi: 10.1016/j.joen.2019.12.012. Epub 2020 Feb 18. PMID: 32081460.
**PORTORREAL, YASIRI**	
*PARPi-FL--a fluorescent PARP1 inhibitor for glioblastoma imaging.	Irwin CP, **Portorreal Y**, Brand C, Zhang Y, Desai P, Salinas B, Weber WA, Reiner T. Neoplasia. 2014 May;16(5):432-40. doi: 10.1016/j.neo.2014.05.005. Epub 2014 Jun 23. PMID: 24970386; PMCID: PMC4198695.
**RAHAMAN, NADIYAH**	
*Molecular Screening for Lynch Syndrome in Young Patients With Colorectal Adenomas.	Mendelsohn RB, Herzog K, Shia J, **Rahaman N**, Stadler ZK, Shike M. Clin Colorectal Cancer. 2017 Sep;16(3):173-177. doi: 10.1016/j.clcc.2017.01.002. Epub 2017 Feb 2. PMID: 28242162.
**RHOOMS, SHAUNA-KAY**	
Dissecting the concordant and disparate roles of NDUFAF3 and NDUFAF4 in mitochondrial complex I biogenesis.	Murari A, **Rhooms SK**, Garcia C, Liu T, Li H, Mishra B, Deshong C, Owusu-Ansah E. iScience. 2021 Jul 16;24(8):102869. doi: 10.1016/j.isci.2021.102869. PMID: 34386730; PMCID: PMC8346666.
An antibody toolbox to track complex I assembly defines AIF's mitochondrial function.	Murari A, **Rhooms SK**, Goparaju NS, Villanueva M, Owusu-Ansah E. J Cell Biol. 2020 Oct 5;219(10):e202001071. doi: 10.1083/jcb.202001071. PMID: 32936885; PMCID: PMC7659709.
Gateways to the Laboratory: How an MD-PhD Program Increased the Number of Minority Physician-Scientists.	Gotian R, Raymore JC, **Rhooms SK**, Liberman L, Andersen OS. Acad Med. 2017 May;92(5):628-634. doi: 10.1097/ACM.0000000000001478. PMID: 28441673.
**ROSENBERG, HALEY**	
*The role of parotid gland irradiation in the development of severe hyposalivation (xerostomia) after intensity-modulated radiation therapy for head and neck cancer: Temporal patterns, risk factors, and testing the QUANTEC guidelines.	Owosho AA, Thor M, Oh JH, Riaz N, Tsai CJ, **Rosenberg H**, Varthis S, Yom SH, Huryn JM, Lee NY, Deasy JO, Estilo CL. J Craniomaxillofac Surg. 2017 Apr;45(4):595-600. doi: 10.1016/j.jcms.2017.01.020. Epub 2017 Jan 31. PMID: 28256385; PMCID: PMC6619497.
*Objective assessment of trismus in oral and oropharyngeal cancer patients treated with intensity-modulated radiation therapy (IMRT).	Owosho AA, Pedreira Ramalho LM, **Rosenberg HI**, Yom SK, Drill E, Riedel E, Tsai CJ, Lee NY, Huryn JM, Estilo CL. J Craniomaxillofac Surg. 2016 Sep;44(9):1408-13. doi: 10.1016/j.jcms.2016.06.008. Epub 2016 Jun 16. PMID: 27377999; PMCID: PMC5279066.
*Primary Ewing Family of Tumors of the Jaw Has a Better Prognosis Compared to Tumors of Extragnathic Sites.	Owosho AA, Ko E, **Rosenberg HI**, Yom SK, Antonescu CR, Huryn JM, Estilo CL. J Oral Maxillofac Surg. 2016 May;74(5):973-81. doi: 10.1016/j.joms.2015.10.029. Epub 2015 Nov 10. PMID: 26679553; PMCID: PMC4850111.
*Osteonecrosis of the jaw in patients treated with denosumab for metastatic tumors to the bone: A series of thirteen patients.	Owosho AA, Blanchard A, Levi L, Kadempour A, **Rosenberg H**, Yom SK, Farooki A, Fornier M, Huryn JM, Estilo CL. J Craniomaxillofac Surg. 2016 Mar;44(3):265-70. doi: 10.1016/j.jcms.2015.12.005. Epub 2015 Dec 20. PMID: 26782845; PMCID: PMC4784099.
**SAVRAN, MICHELLE**	
Characterizing and Quantifying Arbovirus Transmission by Aedes aegypti Using Forced Salivation and Analysis of Bloodmeals.	Miller MR, Sorensen MR, Markle ED, Clarkson TC, Knight AL, **Savran MJ**, Foy BD. Insects. 2021 Mar 30;12(4):304. doi: 10.3390/insects12040304. PMID: 33808172; PMCID: PMC8065531.
**SHAGABAYEVA, LARISA**	
*Drivers of Cost Associated With Minimally Invasive Esophagectomy.	Panda N, **Shagabayeva L**, Comrie CE, Phan N, Moonsamy P, Jeffrey Yang CF, Fernandez FG, Morse CR. Ann Thorac Surg. 2022 Jan;113(1):264-270. doi: 10.1016/j.athoracsur.2021.01.023. Epub 2021 Jan 29. PMID: 33524354.
*Cerebrospinal fluid circulating tumor cells: a novel tool to diagnose leptomeningeal metastases from epithelial tumors.	Lin X, Fleisher M, Rosenblum M, Lin O, Boire A, Briggs S, Bensman Y, Hurtado B, **Shagabayeva L**, DeAngelis LM, Panageas KS, Omuro A, Pentsova EI. Neuro Oncol. 2017 Sep 1;19(9):1248-1254. doi: 10.1093/neuonc/nox066. PMID: 28821205; PMCID: PMC5570249.

*Evaluating Cancer of the Central Nervous System Through Next-Generation Sequencing of Cerebrospinal Fluid.	Pentsova EI, Shah RH, Tang J, Boire A, You D, Briggs S, Omuro A, Lin X, Fleisher M, Grommes C, Panageas KS, Meng F, Selcuklu SD, Ogilvie S, Distefano N, **Shagabayeva L**, Rosenblum M, DeAngelis LM, Viale A, Mellinghoff IK, Berger MF. J Clin Oncol. 2016 Jul 10;34(20):2404-15. doi: 10.1200/JCO.2016.66.6487. Epub 2016 May 9. Erratum in: J Clin Oncol. 2017 Jun 10;35(17):1972. PMID: 27161972; PMCID: PMC4981784.
**SKAKODUB, ANNA**	
*Mechanisms of Ischemic Stroke in Patients with Cancer: A Prospective Study.	Navi BB, Sherman CP, Genova R, Mathias R, Lansdale KN, LeMoss NM, Wolfe J, **Skakodub A**, Kamel H, Tagawa ST, Saxena A, Ocean AJ, Soff GA, DeSancho MT, Iadecola C, Elkind MSV, Peerschke E, Zhang C, DeAngelis LM. Ann Neurol. 2021 Jul;90(1):159-169. doi: 10.1002/ana.26129. Epub 2021 Jun 3. PMID: 34029423.
*Clinical Experience of Cerebrospinal Fluid-Based Liquid Biopsy Demonstrates Superiority of Cell-Free DNA over Cell Pellet Genomic DNA for Molecular Profiling.	Bale TA, Yang SR, Solomon JP, Nafa K, Middha S, Casanova J, Sadowska J, **Skakodub A**, Ahmad H, Yu HA, Riely GJ, Kris MG, Chandarlapaty S, Rosenblum MK, Gavrilovic I, Karajannis MA, Pentsova E, Miller A, Boire A, Mellinghoff I, Berger MF, Zehir A, Ladanyi M, Benayed R, Arcila ME. J Mol Diagn. 2021 Jun;23(6):742-752. doi: 10.1016/j.jmoldx.2021.03.001. Epub 2021 Mar 27. PMID: 33781965; PMCID: PMC8207471.
*Cerebrospinal fluid circulating tumor cells as a quantifiable measurement of leptomeningeal metastases in patients with HER2 positive cancer.	Malani R, Fleisher M, Kumthekar P, Lin X, Omuro A, Groves MD, Lin NU, Melisko M, Lassman AB, Jeyapalan S, Seidman A, **Skakodub A**, Boire A, DeAngelis LM, Rosenblum M, Raizer J, Pentsova E. J Neurooncol. 2020 Jul;148(3):599-606. doi: 10.1007/s11060-020-03555-z. Epub 2020 Jun 6. PMID: 32506369; PMCID: PMC7438284.
*A retrospective, quantitative assessment of disease burden in patients with leptomeningeal metastases from non-small-cell lung cancer.	Nevel KS, DiStefano N, Lin X, **Skakodub A**, Ogilvie SQ, Reiner AS, Pentsova E, Boire A. Neuro Oncol. 2020 May 15;22(5):675-683. doi: 10.1093/neuonc/noz208. PMID: 32352148; PMCID: PMC7229251.
*Tracking tumour evolution in glioma through liquid biopsies of cerebrospinal fluid.	Miller AM, Shah RH, Pentsova EI, Pourmaleki M, Briggs S, Distefano N, Zheng Y, **Skakodub A**, Mehta SA, Campos C, Hsieh WY, Selcuklu SD, Ling L, Meng F, Jing X, Samoila A, Bale TA, Tsui DWY, Grommes C, Viale A, Souweidane MM, Tabar V, Brennan CW, Reiner AS, Rosenblum M, Panageas KS, DeAngelis LM, Young RJ, Berger MF, Mellinghoff IK. Nature. 2019 Jan;565(7741):654-658. doi: 10.1038/s41586-019-0882-3. Epub 2019 Jan 23. PMID: 30675060; PMCID: PMC6457907.
**SLOBOD, ELINA**	
*Retrospective analysis of 18F-FDG PET/CT for staging asymptomatic breast cancer patients younger than 40 years.	Riedl CC, **Slobod E**, Jochelson M, Morrow M, Goldman DA, Gonen M, Weber WA, Ulaner GA. J Nucl Med. 2014 Oct;55(10):1578-83. doi: 10.2967/jnumed.114.143297. Epub 2014 Sep 11. PMID: 25214641; PMCID: PMC4414239.
**SOO-HOO, SARAH**	
Responding to hospital system and student curricular needs: COVID-19 Student Service Corps.	Edelman DS, Desai UA, **Soo-Hoo S**, Catallozzi M. Med Educ. 2020 Sep;54(9):853-854. doi: 10.1111/medu.14243. Epub 2020 Jun 25. PMID: 32418240; PMCID: PMC7276917.
East meets West: the influence of racial, ethnic and cultural risk factors on cardiac surgical risk model performance.	**Soo-Hoo S**, Nemeth S, Baser O, Argenziano M, Kurlansky P. Heart Asia. 2018 Feb 27;10(1):e010995. doi: 10.1136/heartasia-2017-010995. PMID: 29541165; PMCID: PMC5848635.
**SUMNER, RAWLICA**	
*Evaluation of a Commercial Multiplexed Molecular Lower Respiratory Panel at a Tertiary Care Cancer Center.	Cintrón M, **Sumner R**, McMillen T, Mead PA, Babady NE. J Mol Diagn. 2021 Dec;23(12):1741-1748. doi: 10.1016/j.jmoldx.2021.08.002. Epub 2021 Aug 24. PMID: 34438100.
**TEPAN, STEPHANIE**	
*Cancer modeling by Transgene Electroporation in Adult Zebrafish (TEAZ).	Callahan SJ, **Tepan S**, Zhang YM, Lindsay H, Burger A, Campbell NR, Kim IS, Hollmann TJ, Studer L, Mosimann C, White RM. Dis Model Mech. 2018 Sep 27;11(9):dmm034561. doi: 10.1242/dmm.034561. PMID: 30061297; PMCID: PMC6177007.
**VINCENTI, KERRI**	
*Diagnostic value of radiomics and machine learning with dynamic contrast-enhanced magnetic resonance imaging for patients with atypical ductal hyperplasia in predicting malignant upgrade.	Lo Gullo R, **Vincenti K**, Rossi Saccarelli C, Gibbs P, Fox MJ, Daimiel I, Martinez DF, Jochelson MS, Morris EA, Reiner JS, Pinker K. Breast Cancer Res Treat. 2021 Jun;187(2):535-545. doi: 10.1007/s10549-020-06074-7. Epub 2021 Jan 20. PMID: 33471237; PMCID: PMC8190021.
**WOLFE, JULIA**	
**Mechanisms of Ischemic Stroke in Patients with Cancer: A Prospective Study.*	*Navi BB, Sherman CP, Genova R, Mathias R, Lansdale KN, LeMoss NM,* ***Wolfe J*** *, Skakodub A, Kamel H, Tagawa ST, Saxena A, Ocean AJ, Soff GA, DeSancho MT, Iadecola C, Elkind MSV, Peerschke E, Zhang C, DeAngelis LM. Ann Neurol. 2021 Jul;90(1):159-169. doi: 10.1002/ana.26129. Epub 2021 Jun 3. PMID: 34029423.*
*Cancer-Related Ischemic Stroke Has a Distinct Blood mRNA Expression Profile.	Navi BB, Mathias R, Sherman CP, **Wolfe J**, Kamel H, Tagawa ST, Saxena A, Ocean AJ, Iadecola C, DeAngelis LM, Elkind MSV, Hull H, Jickling GC, Sharp FR, Ander BP, Stamova B. Stroke. 2019 Nov;50(11):3259-3264. doi: 10.1161/STROKEAHA.119.026143. Epub 2019 Sep 12. PMID: 31510897; PMCID: PMC6817410.
*Staging identifies non-CNS malignancies in a large cohort with newly diagnosed lymphomatous brain lesions.	Malani R, Bhatia A, **Wolfe J**, Grommes C. Leuk Lymphoma. 2019 Sep;60(9):2278-2282. doi: 10.1080/10428194.2018.1563294. Epub 2019 Jan 10. PMID: 30628502; PMCID: PMC6620158.
*Phase 1b trial of an ibrutinib-based combination therapy in recurrent/refractory CNS lymphoma.	Grommes C, Tang SS, **Wolfe J**, Kaley TJ, Daras M, Pentsova EI, Piotrowski AF, Stone J, Lin A, Nolan CP, Manne M, Codega P, Campos C, Viale A, Thomas AA, Berger MF, Hatzoglou V, Reiner AS, Panageas KS, DeAngelis LM, Mellinghoff IK. Blood. 2019 Jan 31;133(5):436-445. doi: 10.1182/blood-2018-09-875732. Epub 2018 Dec 19. PMID: 30567753; PMCID: PMC6356986.
*Ibrutinib Unmasks Critical Role of Bruton Tyrosine Kinase in Primary CNS Lymphoma.	Grommes C, Pastore A, Palaskas N, Tang SS, Campos C, Schartz D, Codega P, Nichol D, Clark O, Hsieh WY, Rohle D, Rosenblum M, Viale A, Tabar VS, Brennan CW, Gavrilovic IT, Kaley TJ, Nolan CP, Omuro A, Pentsova E, Thomas AA, Tsyvkin E, Noy A, Palomba ML, Hamlin P, Sauter CS, Moskowitz CH, **Wolfe J**, Dogan A, Won M, Glass J, Peak S, Lallana EC, Hatzoglou V, Reiner AS, Gutin PH, Huse JT, Panageas KS, Graeber TG, Schultz N, DeAngelis LM, Mellinghoff IK. Cancer Discov. 2017 Sep;7(9):1018-1029. doi: 10.1158/2159-8290.CD-17-0613. Epub 2017 Jun 15. PMID: 28619981; PMCID: PMC5581705.
**XING, XIN XIN**	
*Surveillance Implications of Recurrence Patterns in Early Node-Negative Esophageal Adenocarcinoma.	Nobel TB, Livschitz J, **Xing XX**, Barbetta A, Hsu M, Tan KS, Sihag S, Jones DR, Molena D. Ann Thorac Surg. 2019 Dec;108(6):1640-1647. doi: 10.1016/j.athoracsur.2019.05.066. Epub 2019 Jul 16. PMID: 31323215; PMCID: PMC6878144.
**YEAHIA, RUBAYA**	
Brain MRI findings in COVID-19 patients with PRES: A systematic review.	**Yeahia R**, Schefflein J, Chiarolanzio P, Rozenstein A, Gomes W, Ali S, Mehta H, Al-Mufti F, McClelland A, Gulko E. Clin Imaging. 2022 Jan;81:107-113. doi: 10.1016/j.clinimag.2021.10.003. Epub 2021 Oct 16. PMID: 34700172; PMCID: PMC8519663.

*Asymmetric Reporting of Harms and Benefits in Randomized Controlled Trials.	**Yeahia R**, Gennarelli RL, Morgan DJ, Korenstein D. J Gen Intern Med. 2021 Aug 11. doi: 10.1007/s11606-021-07056-1. Epub ahead of print. PMID: 34379278.
*Routine Opioid Prescriptions Are Not Necessary After Breast Excisional Biopsy or Lumpectomy Procedures.	Moo TA, Assel M, **Yeahia R**, Nierstedt R, Van Zee KJ, Kirstein LJ, Vickers A, Morrow M, Twersky R. Ann Surg Oncol. 2021 Jan;28(1):303-309. doi: 10.1245/s10434-020-08651-y. Epub 2020 Jun 25. PMID: 32588263; PMCID: PMC7755702.
*Unanticipated Myocarditis in a Surgical Patient Treated With Pembrolizumab: A Case Report.	Nierstedt RT, **Yeahia R**, Barnett KM. A A Pract. 2020 Apr;14(6):e01177. doi: 10.1213/XAA.0000000000001177. PMID: 32132361; PMCID: PMC7101019.
*Quality of Cancer Surveillance Clinical Practice Guidelines: Specificity and Consistency of Recommendations.	Merkow RP, Korenstein D, **Yeahia R**, Bach PB, Baxi SS. JAMA Intern Med. 2017 May 1;177(5):701-709. doi: 10.1001/jamainternmed.2017.0079. PMID: 28319242; PMCID: PMC5590752.
**YUSIM, DIANA**	
HIV Testing Correlates: U.S. and Foreign Born High-Risk Black Heterosexual Men.	Gousse Y, Wilson TE, McFarlane D, Browne RC, Fraser M, **Yusim D**, Stewart M, Salifu MO, Joseph MA. J Immigr Minor Health. 2021 Dec;23(6):1145-1151. doi: 10.1007/s10903-021-01140-8. Epub 2021 Jan 28. PMID: 33507520.
**ZHU, ZHIYING**	
*Use of Traditional Chinese Herbal Medicine Concurrently with Conventional Cancer Treatment Among Chinese Cancer Patients.	Leng J, Lei L, Lei SF, **Zhu Z**, Ocampo A, Gany F. J Immigr Minor Health. 2020 Dec;22(6):1240-1247. doi: 10.1007/s10903-020-01017-2. PMID: 32418000; PMCID: PMC8279391.
*Reproductive factors and lung cancer risk: a comprehensive systematic review and meta-analysis.	Yin X, **Zhu Z**, Hosgood HD, Lan Q, Seow WJ. BMC Public Health. 2020 Sep 25;20(1):1458. doi: 10.1186/s12889-020-09530-7. PMID: 32977782; PMCID: PMC7519481.
*Smoking Among Chinese Livery Drivers.	Leng JC, Lei L, Lei SF, **Zhu Z**, Mo N, Sou B, Mujawar I, Gany F. J Immigr Minor Health. 2019 Apr;21(2):356-363. doi: 10.1007/s10903-018-0740-7. PMID: 29651588; PMCID: PMC6226348.

The total number of peer-reviewed publications listed here is 155, of which 108 were in oncology; however 3 oncology papers each included two students, so that total number of unique papers is 152 and unique papers in oncology is 105.

If a paper is italicized, it means 2 students were co-author; for total # of publications, the italicized paper was included once (with the student for whom it was not italicized, ie the first student listed below).

1 paper: Alija B and Navitski A

1 paper: Skakodub A and Wolfe J

1 paper: Guru N and Huang C
